# Super-Memorizers Are Not Super-Recognizers

**DOI:** 10.1371/journal.pone.0150972

**Published:** 2016-03-23

**Authors:** Meike Ramon, Sebastien Miellet, Anna M. Dzieciol, Boris Nikolai Konrad, Martin Dresler, Roberto Caldara

**Affiliations:** 1 University of Fribourg, Department of Psychology, Rue P.A. de Faucigny 2, 1700 Fribourg, Switzerland; 2 Bournemouth University, Department of Psychology, Talbot Campus, BH12 5BB, Poole, United Kingdom; 3 Cognitive Neuroscience and Neuropsychiatry Section, UCL Institute of Child Health, 30 Guilford Street, WC1N 1EH, London, United Kingdom; 4 Radboud University Medical Centre, Donders Institute for Brain, Cognition and Behaviour, Kapittelweg 29, 6525 EN Nijmegen, The Netherlands; 5 Max Planck Institute of Psychiatry, Kraepelinstr. 2–10, 80804 Munich, Germany; University of Copenhagen, DENMARK

## Abstract

Humans have a natural expertise in recognizing faces. However, the nature of the interaction between this critical visual biological skill and memory is yet unclear. Here, we had the unique opportunity to test two individuals who have had exceptional success in the World Memory Championships, including several world records in face-name association memory. We designed a range of face processing tasks to determine whether superior/expert face memory skills are associated with distinctive perceptual strategies for processing faces. Superior memorizers excelled at tasks involving associative face-name learning. Nevertheless, they were as impaired as controls in tasks probing the efficiency of the face system: face inversion and the other-race effect. Super memorizers did not show increased hippocampal volumes, and exhibited optimal generic eye movement strategies when they performed complex multi-item face-name associations. Our data show that the visual computations of the face system are not malleable and are robust to acquired expertise involving extensive training of associative memory.

## Introduction

Astonishingly high levels of performance may be achieved, seemingly involuntarily, through repeated exposure as demonstrated in the area of face processing. Humans are considered experts at processing faces [1] as they are discriminated and recognized more efficiently than non-face objects of comparable complexity and within-category similarity [2, 3]. The comparably larger detriments for (frequently encountered) faces caused by stimulus inversion (i.e., the face inversion effect; [4–6]), and more efficient processing of faces from the same ethnical background (i.e., the other-race effect, ORE; [7–9]) demonstrate the crucial role of experience for face processing skills [10–12]. However, early proposals suggested that some degree of hard-wiring exists for face recognition [13–15], as supported by more recent studies of its heritability. These studies, involving large cohorts of twins [16, 17], indicate a genetic basis for face recognition, which is a highly specific ability that is uncorrelated with general visual and verbal recognition performance.

Alternatively, extremely proficient performance levels can be acquired deliberately through extensive practice or training. Chess skill, which is attained through extensive, effortful training [18–20], has been associated with differences in perceptual processing [21–25], as well as memory advantages [26]. Mirroring behavioral observations with dog, car, or greeble experts [2, 27–29], recent evidence suggests that face and expert-chess recognition share common processes. Investigating the composite effect for faces and chess boards in expert, novice and recreational chess players, Bogan et al. [30] reported that “[c]hess expertise was positively related to the congruency effect with chess yet negatively related to the congruency effect with faces”. Furthermore, chess ability has been reported to correlate with neural responses in areas recruited in expert object recognition including the fusiform gyrus [31].

Another domain in which skill has been linked to experience and acquired expertise is exceptional memory. Superior memorizers (SMs) utilize mnemonic strategies, usually involving visualization and mental imagery [32]. It is ‘not known whether recall gurus start out with superior memory and attention or gain those skills through practice’ ([33], p.18; see also [34–37]). However, most SMs attribute their superior recall capacities to these highly trained mnemonic techniques [38, 39], which primarily depend on meaningful encoding, mental imagery and prepared retrieval structures [40, 41].

International Memory Championships offer a competitive platform for SMs to demonstrate and compare their exceptional memory skills. These include a number of disciplines designed to test superior memory under various conditions, ranging from memorizing card sequences to abstract forms. One discipline is particularly interesting for the research field of face perception as it probes individuals’ memory skills for stimuli of ‘involuntary’ expertise: associating names with human faces. In the present study we took advantage of the unique opportunity of testing two leading SMs (hereafter referred to as SM1 and SM2). Both have demonstrated a particular proficiency for face-name learning, as evidenced by having repeatedly won the respective discipline at the annual World Memory Championships, and holding world records in all face-related disciplines in memory sports in recent years (http://www.world-memory-statistics.com/discipline.php?id=NAMES15). Furthermore, for many years both have ranked among the top ten contestants in the overall World Ranking of memory sports.

Both cases reported here can be considered as exceptional in comparison to normal controls who are not SMs, as well as representative of the SM population, who achieve astonishing performances through regular training with well-established mnemonic techniques. The SMs studied here are of particular interest as they are particularly proficient in the discipline of face-name learning. In the discipline of non-face picture learning, however, they are not among the top scorers suggesting that their performance may in fact depend on the material to be memorized.

The basis of exceptional memory, as well as the reciprocity between memory and perception have been the addressed by numerous previous studies (for a review see [42]; [43–45]). However, to the best of our knowledge it has not been systematically investigated whether superior memory is associated with differences in visuo-perceptual processing. Therefore, our interest in studying the SMs reported here was twofold.

On the one hand, we sought to determine whether SMs could be considered as ‘Super-recognizers’ [46, 47], i.e., whether or not they present with differential processing and superior recognition of faces as demonstrated by individuals on the high-performing end of the face proficiency continuum. Specifically, Super-recognizers (SRs) are identified as such by their above normal performance on a variety of different tests. These can involve unfamiliar face perception or memory (Cambridge Face Perception Test, CFPT; [48]; Cambridge Face Memory Test, CFMT; [49]; see below for more details), as well as identification of famous individuals by pictures taken long before they were publicly known (Before They Were Famous Test, BTWF; [46]). Their superior ability at upright matching of facial identity [50–52] gives rise to comparably larger a face inversion effect [4], a perceptual phenomenon considered to reflect face-specific processes. In light of these findings, as well as evidence suggesting that visual processing is modulated depending on individuals’ mnemonic goals [53, 54], we investigated whether face processing computations that are considered perceptually rooted are malleable and modulated through explicit training regimes lending to superior memory. Across a range of experiments and recording oculomotor behavior we aimed to ascertain whether two SMs who excel at face related disciplines would exhibit abnormal perceptual face processing as measured by the face inversion and other-race effect.

Furthermore, previous studies indicate that training is associated with structural changes within specific brain regions [55]. The hippocampus is crucially involved in memory processes [56], specifically the formation of associations between distinct items, i.e, relational binding [57]. It plays a prominent role in face-name learning [58–60] and previous studies have reported learning-related functional [37] and structural differences in the hippocampal region of individuals with exceptional memory skills (e.g., [61, 62], however see [37]). We therefore conducted hippocampal volumetric analyses to determine whether extensive training with mnemonic strategies would be associated with increased hippocampal volumes.

## Methods

The project was approved by the institutional Review Committee of the University of Glasgow, Institute of Neuroscience, and the University of Fribourg, Department of Psychology. The procedures were performed in accordance with approved guidelines and regulations; all participants gave written informed consent.

### Subjects

#### Superior memorizers (SMs)

SM1 was 28 and 30 years of age at the time of testing. He is a neuroscientist who has been practicing memory sports since 2002. Among other Guinness world records, he is listed in the 2014 Guinness Book of Records as record holder for memorizing names and faces. In his record performance he memorized 215 German names to the corresponding faces within 15 minutes at the Memoriad in 2015 in Istanbul. He competes in various other memory disciplines and has been ranked in the top ten of the Memory Sports World Ranking list for several years. He teaches mnemonic techniques and credits his superior performance on memory tasks solely to these techniques and training.

SM2 was 33 and 35 years of age at the time of testing. He is a lawyer who first came into contact with memory sports in 2005. He was listed as the international runner up in this sport at the time of testing (currently listed fourth) and has won national and international tournaments. SM2 is memory world record holder in four of the 17 official competition disciplines, thereby holding the most memory world records. For a long time he also held all world records related to names and faces. While many of his other memory records–including three Guinness records–were achieved with memory techniques, according to himself he has always had a very good natural memory for names and faces even before learning about memory techniques. Both SMs reported here provided written informed consent (as outlined in the PLOS consent form) to publish these case details.

#### Control participants

For behavioral Experiments 1–5, we tested samples of Western Caucasian university students who received course credits, or were financially compensated for participation (see below and Table 1 for demographic details). We did not specifically control for age and gender, in keeping with previous studies using similar measures of face processing, which have treated ‘young adults’ aged 17–31 years as a uniform population [63–65]. Similarly, gender differences are (if anything) either minute or absent, and are therefore commonly neglected in oculomotor studies of face perception [66]. For experiments Experiments 1 and 2, which involved face-name learning, German-native speakers were tested as controls for the two German SMs.

**Table 1:** Demographic details for control samples tested for the four tests of face processing and comparison of the hippocampal volumes.

	n	males	Mean age	Age range
Hippocampal volumes	10	10	31±4	25–37
Behavioral experiments				
Face-name learning	21	4	21±3	18–32
Same and other-race face-name learning	16	3	21±3	18–32
Other-race effect	21	3	22±4	18–32
Inversion effect	15	4	27±4	22–35

For measurements of hippocampal volumes, structural neuroimaging data were obtained from age and gender matched members of staff. Oculomotor behavior was acquired from independent control samples for Experiment 1 (n = 15, four males; mean age: 22 years), Experiment 2 (n = 8, one male, mean age: 22 years; one subject’s data for East Asian face-name learning omitted due to technical difficulties during acquisition), and Experiment 4 (n = 15, five males, mean age: 26 years). As there was no indication of differences in oculomotor behavior between SMs and these controls oculomotor data was not registered for Experiment 5.

The data were collected with the understanding and written consent of each subject; the experiments conform to The Code of Ethics of the World Medical Association (Declaration of Helsinki) and comply with the ethical guidelines of the universities where testing took place. All individuals depicted in the figures exemplifying stimuli used provided written informed consent (as outlined in the PLOS consent form) to publish images of their likeness.

Individual SM’s behavioral results were compared to those of the respective control groups according to Crawford and Garthwaite [67]. This procedure has been developed to estimate the abnormality of individuals’ test scores relative to control samples; it is most useful given modest sample sizes, but can be used with data from control samples of any size.

### Imaging procedures

For the measurement of hippocampal volumes, T1-weighted three-dimensional MPRAGE images were collected with a 3T Siemens Trim Trio scanner (Siemens Medical, Munich, Germany). 192 1mm-thick sagittal slices were acquired with a field of view of 256 mm^2^, leading to a voxel size of 1×1×1 mm^3^. Echo time was 2.52 ms; repetition time, 1.9 ms; flip angle, 9 degrees. Hippocampal volumes were measured by one of the authors, AD, using BRAINS2 [68] while blind to subject identity. First, the hippocampus was outlined on every third slice in the sagittal plane, starting in the most lateral section and continuing medially until it was no longer visible. Next, markers representing the outlines made on the sagittal sections were displayed in the coronal plane. This approach helped to delineate the hippocampal boundaries on the coronal sections, especially with respect to the dorsal border with the amygdala. The hippocampus was then segmented in every coronal section, following a protocol published previously [69]. The hippocampal volumes were calculated by summing the areas of the coronal outlines. For each participant, a second measurement was obtained by the same rater, and the average was used for further analysis. Intra-rater reliability of the two measurements was deemed ‘excellent’ (intra-class correlation coefficients: left hippocampus, .910; right hippocampus, .952). Intracranial volumes were obtained automatically using the ‘*New segment’* procedure in SPM8 (http://www.fil.ion.ucl.ac.uk/), running in Matlab (The MathWorks, Inc). To adjust for the brain size, the hippocampal volumes were then divided by the intracranial volumes. The volumetric analyses involved two-tailed testing to assess any potential differences between SMs and controls.

### Behavioral testing

We performed five (sets of) experiments to assess SMs’ face memory and perceptual face processing skills. To confirm their superior face-name association learning skills as demonstrated by their unique track record in memory sports, we first tested face-name learning situations as they occur in international memory competitions, with numerous uncontrolled stimuli and names presented simultaneously (Experiment 1; see Results). Next, subjects learned the names of individually presented same, and other-race faces, using stimuli which were more controlled (available external and non-facial information, low-level visual properties; Experiment 2), to ensure that their superior performance would also be observed under such experimental conditions. Furthermore, we aimed to determine whether an own-race superiority would be observed for associative face-name learning, in order to investigate potential recognition and perceptual processing differences with further experiments.

As mentioned, both SMs tested here attribute their superior face-name memory performance to the deliberate training of mnemonic techniques. Therefore, the remaining experiments intended to evaluate this claim using previously reported or classical paradigms to investigate recognition and perceptual processing of faces. First, given previous reports of individuals with extraordinary face processing skills, we aimed to determine whether the SMs would be considered as ‘super-recognizers’ using three previously reported tests of face discrimination and recognition [46, 47]. These tests (Experiment 3; detailed below) are considered to tap into perception and memory for unfamiliar faces (CFPT, [48]; CFMT, [49]), as well as identification of famous individuals by pictures taken before they were famous [BTWF; [46]. It should be noted, however, that merely the CFMT is accepted as a tool for identifying SR as well as developmental prosopagnosic individuals. The “large variability in control performance on the CFPT results in a large standard deviation, making significant differences on single-case analyses near impossible to achieve” [50]. Additionally, the correlations obtained between BTWF and CFMT in the only study reporting BTWF control data [46] “suffer from a sampling error [and therefore] should be seen as tentative”[50].

Finally, two further experiments aimed at tapping into perceptual processing of faces, involving old/new decisions of same and other race faces (Experiment 4) and a delayed matching task with upright and inverted faces (Experiment 5).

In line with their successful application of memory techniques, we anticipated that SMs would generally exhibit superior performance for tasks involving learning of face-name associations. On the other hand, in line with our hypothesis that SMs are not Super-recognizers, we expected them to perform similarly to normal subjects and below the performance of Super-recognizers as described previously. Likewise, we expected to observe an advantage for processing same- over other-race faces, and upright over inverted faces, as exhibited by normal observers. With the exception of Experiment 3, in which previously reported tests that are made available for research purposes [46] were used, stimuli were specifically tailored to meet the requirements of the task as described in the following.

#### Experiment 1: Face-name association learning with naturalistic images and external cues

To validate the expected performance differences between SMs and controls, the first experiment was designed to approximate the face-name learning task applied in international memory competitions. In keeping with this procedure, we selected stimuli that were not controlled with regards to superficial image properties and availability of external/non-facial information. However, in an attempt to minimize floor effects in controls, the face-name pairings were viewed freely with no time constraints (participants’ learning times were recorded). Ninety-six full frontal color photographs were selected from those made available by various Olympic teams’ websites. These were cropped to the same size and randomly assigned German first and last names (face-name associations identical for all participants). Four different ‘grids’ of faces with their respective names were created, encompassing 1395 x 1048 pixels (150 dpi). Each grid depicted 12 females and 12 males. Subjects viewed each of the four grids freely with no time constraints (random order of presentation). After all grids had been learned once, this was repeated a further two times, i.e., each grid was learned three times in total. Stimulus presentation and response registration was controlled using Matlab (The MathWorks, Inc.) and the Psychophysics Toolbox extensions [70, 71]. Upon completion of the last learning session, and after a two minute delay, participants received four sheets depicting the learned grids, albeit without names. They had unlimited time to write down as many first and/or last names as they could recollect. Subjects received .5 points for each name recalled correctly (first or last); the maximum score that could be achieved was thus 96 points.

#### Experiment 2: Western Caucasian (WC) and Eastern Asian (EA) face-name learning

The second experiment was designed to test whether SMs would show an advantage for processing own over other-race faces in the realm of an explicit memory task, i.e., not in the commonly used old/new recognition tasks (see below; [72]). Contrary to Experiment 1, here more controlled stimuli were used (for examples see Results), which were further presented individually. Two sets of stimuli (same and other race, i.e., WC and EA) were created from a larger, continuously expanding database of 3D face models developed at the Institute of Neuroscience and School of Psychology at University of Glasgow, UK. Stimuli from this database have been used to address a range of different research questions and are readily perceived as WC or EA, respectively (see e.g., [73–75]). Each stimulus set consisted of images of 50 identities (25 females), which were full-frontal color images rendered from the identities’ original 3D face models using 3D Studio Max. The images were cropped of external features and placed on a grey background canvas encompassing 600 x 800 pixels. All faces were normalized for feature location, as well as luminance and contrast; all faces were randomly assigned entirely new German first and last names (identical across subjects). Participants completed face-name association learning tasks for WC and EA faces, respectively, on two separate days (order randomized; intervals between testing identical for controls and SMs). Stimulus-name pairs were presented without time constraints, but subjects were informed that they would see each pair only once; subsequent trials were initiated by subjects pressing the space bar. After all 50 pairs were presented the previously learned images were presented at random without names while the experimenter recorded subjects’ verbal responses. Stimulus presentation and response time registration was controlled using Matlab (The MathWorks, Inc.) and the Psychophysics Toolbox extensions [70, 71]. Subjects received .5 points for each name recalled correctly (first or last); the maximum score that could be achieved for both Western Caucasian and East Asian face-name learning was 50 points.

#### Experiment 3: Tests of superior face recognition

To determine whether our SMs could be considered Super-recognizers we employed three tests that have been utilized in healthy and abnormal populations to assess individuals’ face processing skills: the (long version of the) Cambridge Face Memory Test (CFMT) and Cambridge Face Perception Test (CFPT) [47–49], and the Before They Were Famous Test (BTWF) [46], which are available free of charge when used for research purposes. The CFMT involves learning of six unfamiliar male faces from three different views, the recognition of which is then tested in a three-alternative forced-choice task with increasing difficulty as the test proceeds. This test has been used in more recent studies to identify both SR and developmental prosopagnosics [50–52]. The CFPT is a test of face matching based on perceived similarity, for both upright and inverted faces. Subjects are required to ‘sort a set of six frontal views of faces by similarity to a target face shown from a three-quarter view’ [46]. The frontal view face stimuli were created by morphing the target face with six other faces, in varying proportion to create stimuli varying in similarity to the target face. Finally, in the BTWF test observers are presented 56 photographs of famous individuals, depicting said individuals before they were famous (often as children), which they are required to identify by name or other uniquely identifying personal information. As noted above, although the most reliable and commonly accepted means of identifying SRs remains the CFMT [50], we tested SM1 and SM2 using the CFPT and BTWF.

#### Experiment 4: Other-race effect (ORE)

The fourth experiment has been reported previously as a sensitive measure for demonstrating the well-established ORE in the realm of an old/new decision task [72]. In this setting the ORE manifests as subjects’ comparably higher proficiency at recognizing faces of their own (as compared to another) race (as tested with two groups of observers and stimuli, respectively). Thus, this experiment was applied to ascertain whether SMs present with an ORE, as robustly observed in observers who do not practice mnemonic techniques (see Results for examples of stimuli; a detailed description of the stimuli and experimental procedure is provided elsewhere [72]). Subjects were presented four learning blocks, each of which was followed by a recognition block. Each block consisted of images of faces of same-race (WC faces, taken from [76]), or other-race individuals (EA faces, taken from [77]); the same procedure as follows was adopted independent of stimulus race. Fourteen identities were presented in each learning block. Subsequent recognition blocks involved presentation of 28 identities (14 old/new). Importantly, the identities learned were depicted using different images than those used for recall in the recognition blocks (the same identity displayed different facial expressions; for examples see Results) to circumvent participants employing image-based learning strategies. Subjects were required to distinguish previously learned (‘old’) from new identities. Stimulus presentation and response registration was controlled using Matlab (The MathWorks, Inc.) and the Psychophysics Toolbox extensions [70, 71].

#### Experiment 5: Face Inversion Effect (FIE)

This final experiment involved a perceptual matching task devoid of an explicit long-term memory component. It was designed to assess whether SMs would show a decrease in face matching performance that is attributed to deficient perceptual encoding of stimuli when presented in an uncommon orientation. This phenomenon—referred to as the face inversion effect [4]—is reduced or absent in developmental and acquired prosopagnosia [47, 78, 79], and its magnitude correlates with processing of facial identity [46]. Thus, here we aimed to determine whether SMs would exhibit a larger FIE as compared to controls. The stimuli were taken from the stimulus set created elsewhere [80]; these consisted of images of 20 females converted to greyscale, cropped of external features and placed on a 242 x 342 pixel canvas with grey background. Additionally, for each image, a noise mask with the same power spectrum as the original image was created (for examples see Results). Subjects performed a delayed match-to-sample, two-alternative forced-choice task, implemented with an experimental procedure similar to that employed previously [46, 81]. On each trial a target stimulus was presented centrally for 150 ms; after a 500 ms blank, the target’s mask was presented for 200 ms (target faces and mask encompassed ~7 and 8 degrees of visual angle in height). After a 1000 ms blank two juxtaposed probes (~9 degrees of visual angle in height) were presented for 500 ms. Subjects responses were recorded 2500 ms from the probes’ onset; trials were separated by a 1000 ms inter-trial interval. The experiment comprised 760 trials (20 identities, paired with each other one; each combination presented twice to control for response side), and was completed twice by each subject, on two separate days. For these testing sessions, participants received different instructions while matching the identical stimuli (order randomized across subjects): irrespective of the stimulus orientation (which varied randomly across trials), they were told to utilize either information located in the lower visual field, or the eyes. This was done because after initial testing of SM1, he commented that irrespective of stimulus orientation he had focused on the bottom of stimuli presented. SM2 on the other hand reported having focused on the eyes across orientations. For this reason, on the following testing day, we requested both to utilize the respective other strategy and adopted the same procedure for control subjects (interval between testing identical for controls and SMs). Stimulus presentation and response registration were controlled using Eprime. Contrary to Experiments 2 and 4, which involved stimulus presentation blocked by condition (i.e., race), here orientation varied randomly on a trial-by-trial basis. Therefore, per behavioral measure, analyses were carried out on the difference between upright and inverted face processing observed for SMs as compared to control subjects.

### Eye tracking

For Experiments 1, 2 and 4 oculomotor data was acquired in independent groups of controls and SMs. Across experiments, saccades and fixations were determined using a custom algorithm using the same filter parameters as the EyeLink software (saccade velocity threshold = 30°/sec; saccade acceleration threshold = 4000°/sec2) and merging fixations close spatially and temporally (<20ms, < .3°).

Eye movement patterns were compared at a general level between SMs and controls. For single faces studies (i.e., Experiments 2, 4), the patterns were also compared to prototypical patterns corresponding to local vs. global information sampling strategies. Finally, the number of fixation clusters was considered a proxy of the number of key regions attended to.

Fixation distribution maps, weighted by fixation duration, were extracted individually for each observer. The fixation maps of each of the SMs were correlated with those of each of the controls. Similarly, the fixation maps of the controls were correlated with those of all the other controls. Since correlation coefficients are not additive, they must be z-normalized [82] before performing statistical analyses. We thus normalized the obtained correlation coefficients by using Fisher’s transform $\text{Z}\;=\;0.5\cdot \;{log}_{e}\left|\frac{1+r}{1-r}\right|$. We then applied a bootstrapping procedure (10000 resamples) on the obtained Fisher transformed correlation coefficients in order to obtain the distributions of fixation pattern correlations between on the one hand each SM compared to the controls, and on the other hand each control compared to the other controls.

We also correlated the SMs’ fixation patterns to simulated prototypical patterns corresponding to global vs. local information sampling strategies (fixations on the center of the face, vs. on the eyes and/or mouth, respectively; [83]). The prototypical patterns were created by generating 2000 normally distributed random fixations (.5 degree of visual angle as standard deviation) around either the eyes and mouth for the local prototype, or around the center of the face for the global prototype.

Finally, the number of significant fixation clusters (*p <* .*05*) for each observer was determined with the *i*Map toolbox (version 2.1; [84]). *i*Map identifies fixation clusters in the stimulus space using a robust statistical approach correcting for multiple comparisons, by applying a one-tailed Pixel test [85]. *i*Map2.1 does not take into account variability across observers [86], which is a valid approach here given the fact that we are considering the number of fixation clusters at an individual level. In experiment 1, the number of significant clusters was determined for each grid stimulus independently as there was no reason to expect similar patterns between grids. The number of significant clusters was then averaged across grids.

## Results

### Hippocampal volumes

**Fig 1:**
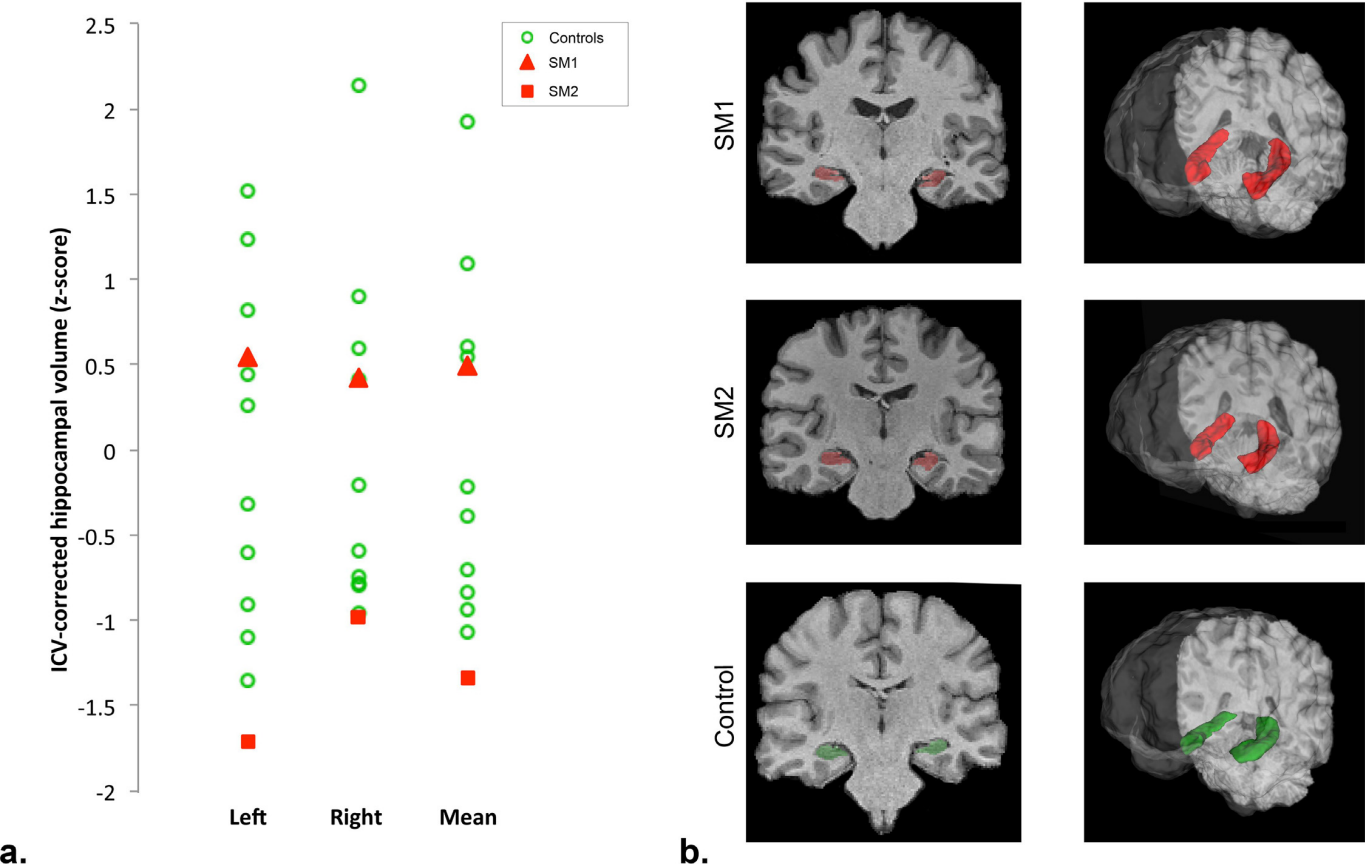
Hippocampus volumetry in SMs and controls. **a.** ICV-corrected hippocampal volumes of SMs and controls. Z-scores were calculated based on means and standard deviations of 10 control participants. **b.** Hippocampi of SM1 and SM2 (red) and one representative control subject (green) displayed on a coronal slice, as well as in 3D.

The hippocampal volumes of all participants are shown in Fig 1. Mean and unilateral intracranial volume (ICV) corrected hippocampal volumes of SM2 were significantly below those of control participants (mean, t(9) = -4.24, *p =* .*002*; left t(9) = -5.39, *p <* .*001*; right t(9) = -3.09, *p =* .*013*, one sample t-tests, two-tailed). Hippocampal volumes of SM1 did not differ from the controls’ (mean, t(9) = -1.55, *ns*; left, t(9) = -1.71, *ns*; right, t(9) = -1.34, *ns*; one sample t-tests, two-tailed). Thus, our results clearly do not show a positive association between hippocampal volume and exceptional memory skills for face-name associations.

### Behavioral testing

#### Experiment 1: Face-name association learning with naturalistic stimuli

**Fig 2:**
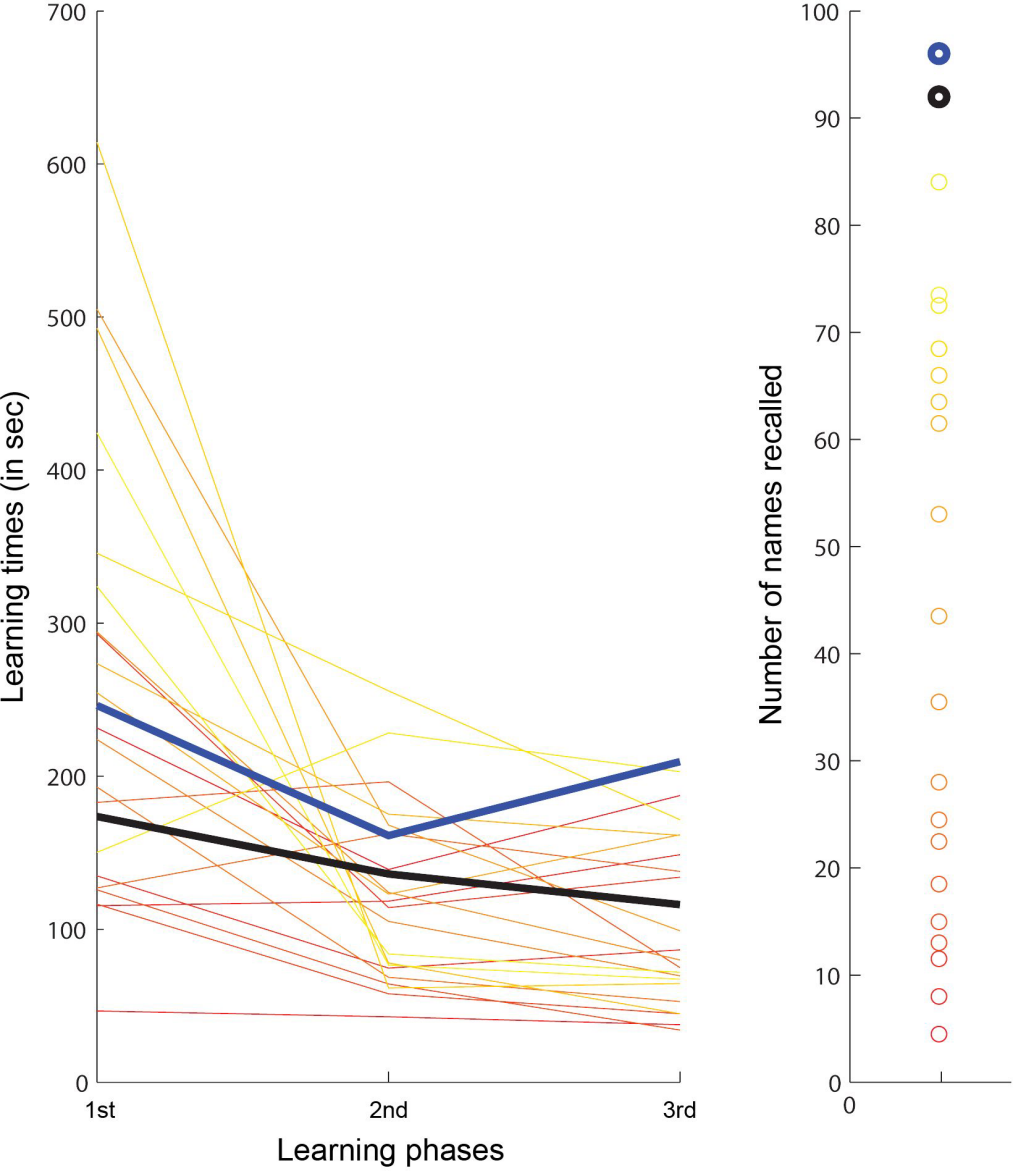
Individual subjects’ results for Experiment 1: Face-name association learning with naturalistic stimuli. Subjects’ learning times (left) and overall recognition scores (right; subjects received .5 points per correctly recalled name). Individual controls are depicted in yellow to red colors, in order to relate their learning times to the respective scores obtained; SMs’ performance is displayed as black (SM1) and blue (SM2) lines and circles, respectively.

Table 2 and Fig 2 summarize the results observed for SMs and control subjects. Of the 96 possible points, SM1 and SM2 scored 92 and 96, respectively, which was significantly better than controls (t(19) = 2.01, *p =* .*03*, and t(19) = 2.16, *p =* .*02*). This superior performance cannot be attributed to differences in learning times, as for each learning phase, SMs’ learning times did not differ significantly from the time spent by controls (SM1: t(19) = -.54; t(19) = .27, t(19) = .25; SM2: t(19) = -.09; t(19) = .25; t(19) = 1.95, *ns* per learning phase and SM subject). Note that controls’ behavior was characterized by a large degree of variability, both in terms of learning times and recall performance.

**Table 2:** Results for Experiment 1: Face-name association learning with naturalistic stimuli.

		Learning times			
	Score	1^st^ phase	2^nd^ phase	3^rd^ phase	Total
**Controls**	37.5±26.5	260±149	120±59	102±54	161±87
**SM1**	92	174	136	116	142
**SM2**	96	246	161	210	206

Analyses of oculomotor behavior revealed highly variable fixation patterns for SM vs. controls, or controls vs. controls. SMs’ fixation patterns were not less correlated with the controls’ than the controls between themselves (SM1 vs. controls: .34±.06; SM2 vs. controls: .27±.06; controls vs. controls: .19±.02). However, SMs exhibited a larger average number of fixation clusters than controls (SM1: 20; SM2: 19.5; controls’ range: 11–18), indicating that SMs attended to significantly more faces than the controls.

#### Experiment 2: Western Caucasian and Eastern Asian face-name learning

**Fig 3:**
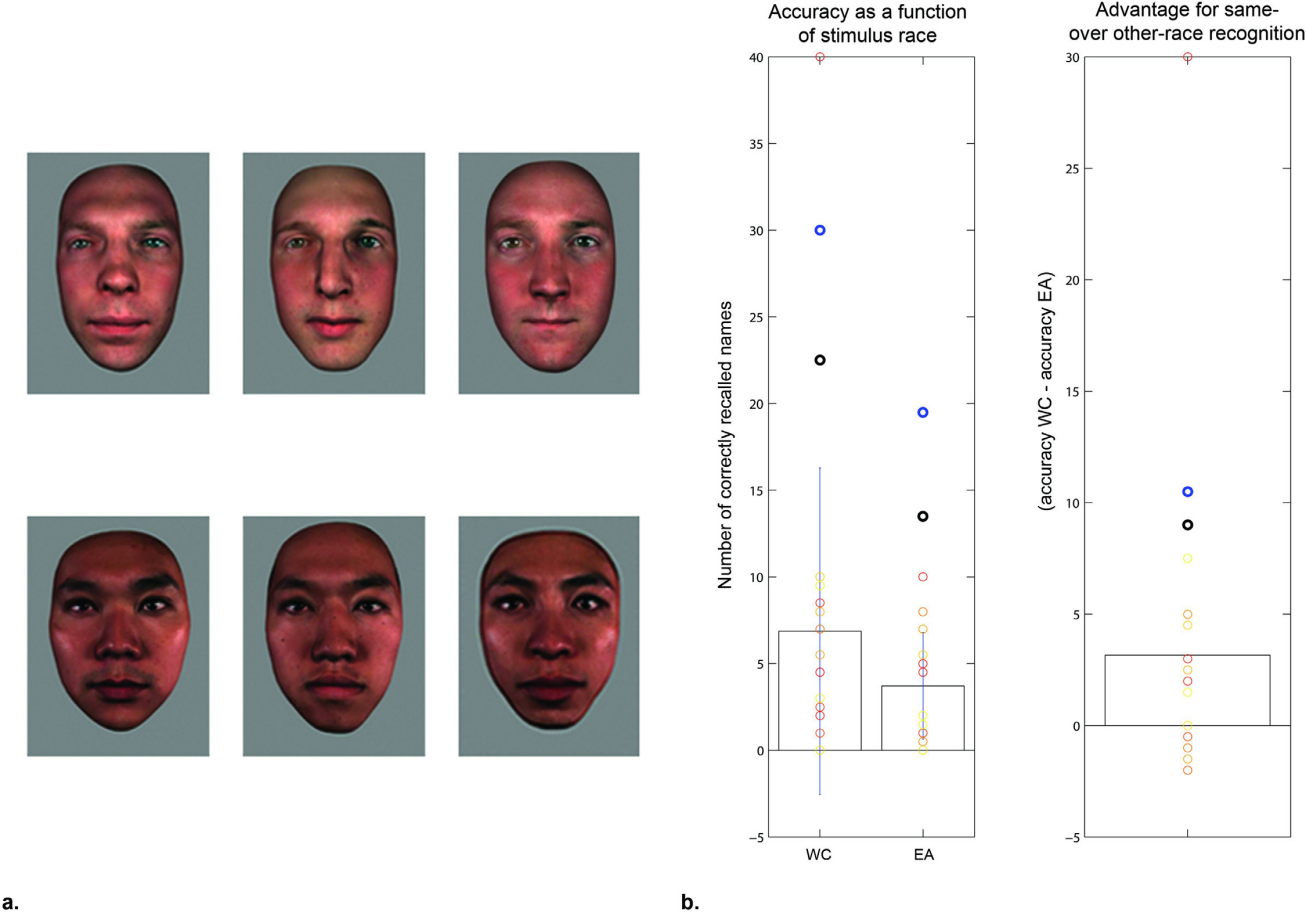
a. Examples of stimuli and b. results for Experiment 2: Western Caucasian and Eastern Asian face-name learning. Individual subjects’ performance (number of correctly recalled first and/or last names) plotted as circles on top of the group (bars represent standard deviations); dark circles demonstrate SMs’ performance (SM1: black; SM2: blue).

Table 3 and Fig 3B summarize the results observed for SMs and control subjects. Statistical tests were performed to test the hypothesis that own-race face processing would be more efficient (i.e., higher recognition scores, shorter learning times). Controls’ scores (i.e. number of correctly recalled names) were non-significantly higher for WC as compared to EA faces, t(15) = 1.65, *p =* .*06*. This should be attributed to the floor effect observed for WC face stimuli in over half of the subjects. Contrariwise, subjects who achieved at least 8 points for WC faces exhibited inferior performance for EA faces, as well as shorter learning and/or recognition times for own-race compared to other race faces. Controls’ learning and correct recognition times were not shorter for WC as compared to EA faces (t(15) = .19, and t(12) = -1.26, *ns*). Both SMs correctly named significantly more WC than EA face stimuli (SM1: t(98) = 2.12, *p =* .*02*; SM2: t(98) = 2.18, *p =* .*02*). Neither of the SMs’ learning times were longer for EA as compared to WC faces (SM1: t(98) = -2.30, *p =* .*99*; SM2: t(98) = -1.16, *p =* .*88*); the same held for their recognition times (SM1: t(43) = -.56, *p =* .*71*; SM2: t(50) = -1.02, *p =* .*84*). Thus both SMs showed a clear advantage for learning and recalling face-name associations of same, as compared to other-race faces. The lack of such a difference on the group level for controls should be attributed to over half of the controls exhibiting floor effects.

**Table 3:** Results for Experiment 2: Western Caucasian and Eastern Asian face-name learning.

	Western Caucasian			East Asian		
	Score	Learning time	Recall time	Score	Learning time	Recall time
**Controls**	6.9±9.4	28±23	15±6	3.7±3.1	28±19	18±8
**SM1**	22.5	17±6	23±22	13.5	15±6	20±13
**SM2**	30	38±19	24±22	19.5	33±21	19±11

With regards to oculomotor behavior, SMs’ fixation patterns were not less correlated with the controls’ than the controls between themselves. This held for both learning of WC face-name associations (SM1 vs. controls: .77±.06; SM2 vs. controls: .88±.11; controls vs. controls: .67±.03), as well as EA ones (SM1 vs. controls: .73±.07; SM2 vs. controls: .90±.09; controls vs. controls: .69±.06). The number of fixation clusters exhibited across control subjects ranged between 1 and 4 for both WC and EA faces, within which SMs’ fell (see S1 Table). The individual fixation maps further confirm the absence of differences between controls’ and SMs’ fixation patterns (see S1 Fig). Controls displayed individual variability in idiosyncratic sampling strategies, from global information sampling strategies characterized by fixations on the center of the face, to local strategies typified by fixations on the eyes and mouth ([87]; see S1 Table). Although less obvious for EA as compared to WC faces, SM1 showed a tendency to adopt a local, rather than a global face information sampling strategy, while SM2 seemed to adopt a more global strategy.

#### Experiment 3: Tests of superior face recognition

For the long version of the CFMT SM1 and SM2 achieved 62 and 67 correct, respectively. These scores are well below the previously used cut-off of (90/102; i.e., two standard deviations above the control average; [46, 47, 50–52]). On the BWTF task SM1 and SM2 recognized 3 and 19 individuals, respectively. For the CFPT, the number of errors for upright vs. inverted face matching exhibited by SM1 was 46 vs. 54, while SM2 made 18 vs. 50 mistakes. The SMs’ scores are either within the normal range (SM1) or the high end (SM2) of previously reported controls (n = 26, mean upright error ± SD: 35.4±12.9; [47]).

#### Experiment 4: Other-race effect (ORE)

**Fig 4:**
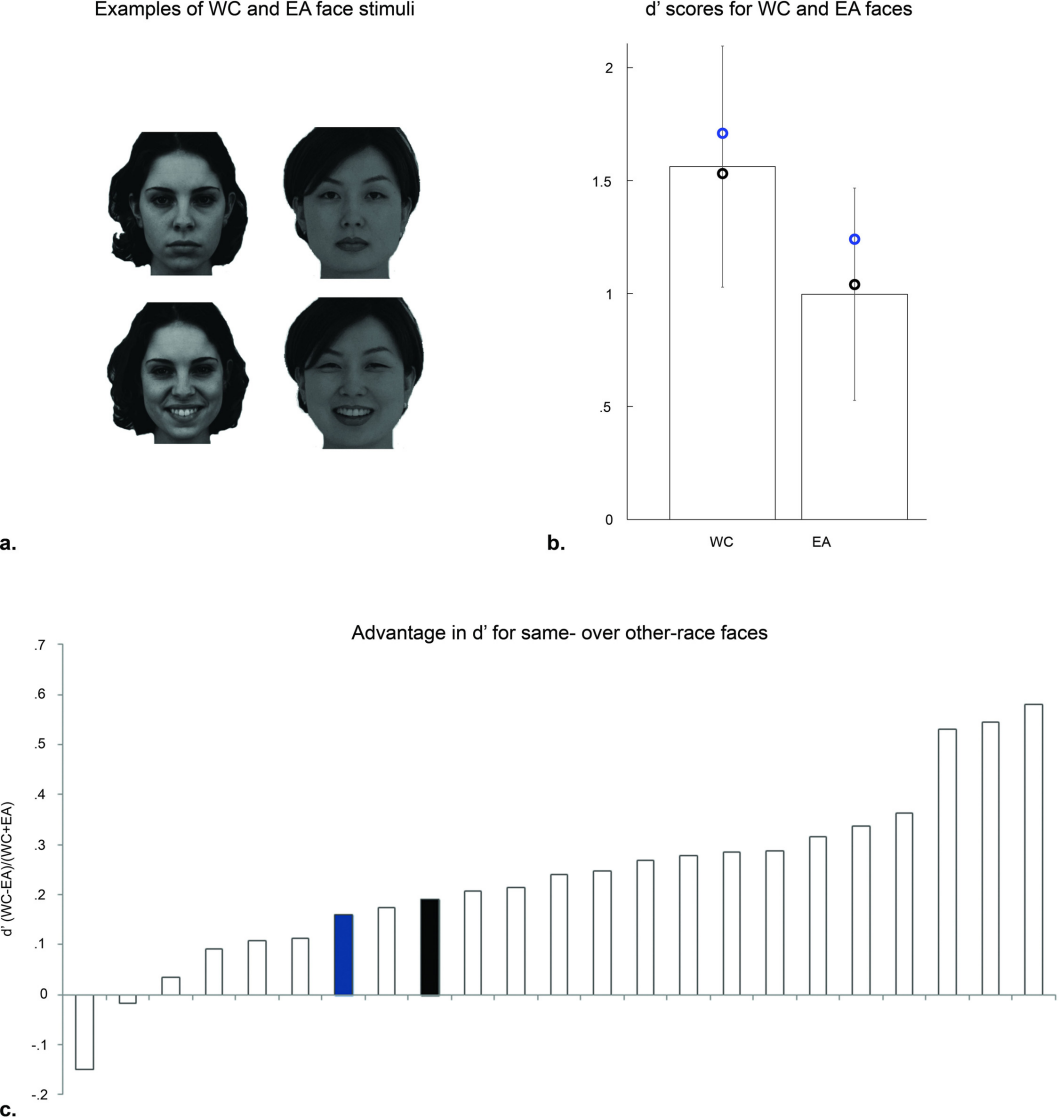
Examples of stimuli and results for Experiment 4: Other-race effect (ORE). **a.** Examples of stimuli presented. During encoding subjects were presented images of WC and EA identities, which were presented depicting a different facial expression during recognition. **b.** Controls’ mean d’ (bars represent standard errors) per race with SMs’ scores (SM1: black; SM2: blue) superimposed. **c.** Individual subjects’ d’ advantage for recognition of same- over other-race faces (ranked normalized difference scores). All subjects, including SMs showed superior performance for old/new decisions of own-race faces.

Table 4 and Fig 4 summarize the results observed for SMs and control subjects. Controls’ RTs did not differ as a function of stimulus race, t(20) = -1.43, *p =* .*17*, but their d’ scores were significantly higher for WC as compared to EA stimuli, t(20) = 6.53, *p <* .*001*. Although SM1’s RTs were significantly longer than those of controls for WC stimuli, t(19) = 2.55, *p =* .*02*, the difference in RTs for WC and EA stimuli was of comparable magnitude as the non-significant RT difference (see above) observed for controls, t(19) = -1.75, *p =* .*10*. Comparing SM1’s RTs across conditions revealed no significant difference, t(38) = 1.12, *p =* .*27*. SM2’s RTs did not differ significantly from those exhibited by controls, irrespective of race of stimuli (WC: t(19) = 1.35, *p =* .*19*; EA: t(19) = -.24, *ns*). Most importantly the difference in d’ exhibited by both SMs was comparable to the d’ difference observed for control subjects (SM1: t(19) = -.38, *ns*; SM2: t(19) = .23, *ns*).

**Table 4:** Results for Experiment 4: Other-race effect.

	d’			RT		
	WC	EA	$\frac{\left(WC-EA\right)}{\left(WC+EA\right)}$	WC	EA	$\frac{\left(WC-EA\right)}{\left(WC+EA\right)}$
Controls	1.56±.53	1.00±.47	.24±.18	1.74±.70	1.91±.76	-.04±.13
SM1	1.53	1.04	.19	3.57	2.87	.11
SM2	1.71	1.24	.16	2.71	1.72	.23

Paralleling the findings of Experiment 2, SMs’ fixation patterns were not less correlated with the controls’ than the controls between themselves (SM1 vs. controls: .77±.07; SM2 vs. controls: .72±.05; controls vs. controls: .64±.03). As for Experiment 2, controls’ strategies varied in the extent to which their information sampling style could be described as local, as opposed to global (see S1 Table), with SM1 and SM2 exhibiting a more local and global information sampling strategy, respectively (see also S2 Fig).

#### Experiment 5: Face inversion effect

**Fig 5:**
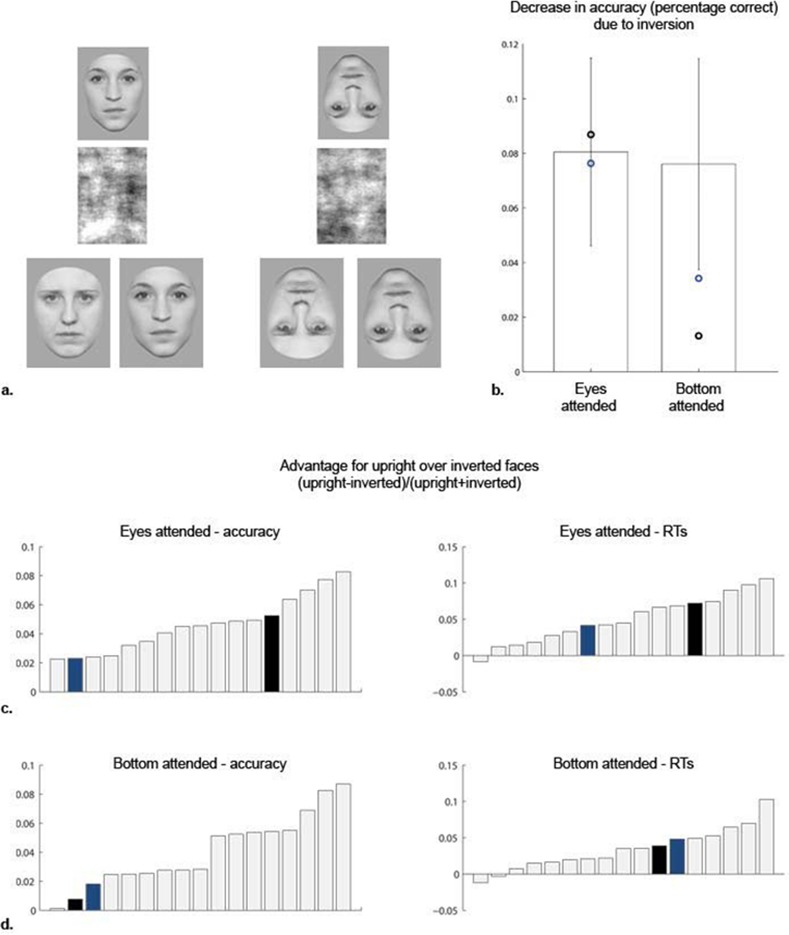
Experimental design and results for Experiment 5: Face inversion effect (FIE). **a.** Examples of stimuli presented on a given trial (see Methods). **b.** Controls’ average decrease in accuracy related to inversion as a function of face part attended. Individual subjects’ accuracy and RT advantage for upright over inverted face matching when attending **c.** the eyes and **d.** the mouth. Note that SMs’ performance decreases (SM1: black; SM2: blue) were not significantly different from those observed for controls.

Table 5 and Fig 5 summarize the results observed for SMs and control subjects. Irrespective of region attended (eyes vs. bottom part of the face), as well as behavioral measure considered, performance detriments related to stimulus inversion did not differ between SMs and control subjects. For matching of the eye region, SM1’s and SM2’s inversion effects did not differ significantly from those observed in controls (accuracy: t(13) = .18, and t(13) = -.12, *ns*; RTs: t(13) = .64, and t(13) = .23, *ns*). The same was observed for matching of bottom face parts, where both SMs’ inversion effects did not differ significantly from those exhibited by controls’ (accuracy: (t(13) = -1.57, *p =* .*07*, and t(13) = -1.05, *p =* .*16*; RTs: (t(13) = -.30, t(13) = .72, both *ns*).

**Table 5:** Results for Experiment 5: Face inversion effect.

	upright		inverted		$\frac{\left(upright-inverted\right)}{\left(upright+inverted\right)}$	
Eyes matching	accuracy	RT	accuracy	RT	accuracy	RT
**Controls**	92.63±4.47	591±96	84.58±4.75	653±119	.0456±.0200	.0481±.0339
**SM1**	87.11	596	78.42	689	.0525	.0721
**SM2**	84.21	511	76.58	584	.0475	.0665
Bottom matching						
**Controls**	91.10±3.74	597±105	83.50±5.84	642±129	.0443±.0241	.0342±.0302
**SM1**	86.05	743	84.74	775	.0077	.0208
**SM2**	96.32	765	92.89	842	.0181	.0480

## General Discussion

In the present study we tested face processing and memory of two superior memorizers (SMs), who regularly excel in international memory competitions and have particularly demonstrated unique performances in the discipline of face memory, as compared to learning of non-face visual material. Although here we tested only two single cases, the SMs reported here credit their memory skills to deliberate training in mnemonic strategies employed by SMs in general, and share several world records in face-name association memory. Several studies have indeed demonstrated that mnemonic strategies can considerably improve face-name association learning [88–90]. We sought to determine whether superior face-name association learning abilities would be observed alongside differences in perceptual strategies of face processing. This is of particular interest given recent findings suggesting that individuals’ mnemonic goals modulate visual processing [53, 54]. Furthermore, differences in perceptual processing and memory advantages have been reported for other fields of expertise, e.g. expert chess players [21–26]. Previous investigations involving SMs have classically focused on identifying intellectual, anatomical and functional correlates of superior memory (e.g., [37, 39]). Our study provides novel insights in the field, by showing that superior memory does not rely on fundamentally different visual strategies for faces.

Our results demonstrate that neither of the SMs tested here can be considered a ‘Super-recognizer’ [46, 47, 50–52]. Relative to control subjects, SMs’ superior memory performance for name-face associations was observed in the absence of significantly increased hippocampi or fixation pattern differences during tasks of face perception. Finally, extensive and efficient usage of mnemonic techniques did not abolish commonly reported perceptual impairments of face processing (other-race effect, inversion effect). This contrasts recent evidence suggesting that holistic face processing may be modulated by cognitive training in individuals with developmental, but not acquired prosopagnosia [91, 92]. We observed that SMs’ eye movements differed from those of controls only in the context of a task involving memorization of face-name association for multiple, simultaneously presented items. Together, these findings lend support to the idea that face processing abilities are at least to a certain extent hard-wired and robust to training.

### Neurofunctional relationship between memory and perception

Several studies have reported neuroanatomical changes induced by cognitive training (for a review see e.g., [55]), including those observed in the hippocampi of London taxi drivers [61]. The hippocampus is crucially involved in memory processes [56] and particularly face-name learning [58–60]. Various studies indicate that it is critical for domain-general relational binding (i.e., the formation of associations among distinct items; for a review see [57]), and abnormal hippocampal development is associated with face learning impairments in cases of developmental amnesia [93]. Therefore, we sought to determine whether the SMs tested here would present with increased hippocampal volumes.

Supporting previous studies which reported no differences in hippocampal volume in SMs [37], and despite our SMs reporting excessive training in a spatial learning strategy (i.e., the method of loci), they did not present with increased overall hippocampal volumes. Note that Maguire et al. [61, 62] reported differential effects of navigational expertise on subsections of the hippocampus in London taxi drivers. Further studies with larger number of SMs are required to address the question whether memory skill is associated with systematic differences in sub-regions of the hippocampus.

Our data offer the view that rather than coarse structural differences in the hippocampus, superior memorizers and controls will likely differ on the functional level of distributed networks, involving prefrontal and medial temporal lobe structures, which operate in concert with those involved in visual processing [37, 94, 95]. Recent evidence suggests that neural signatures of vividness and memorability can be observed within structures that receive extensive inputs from prefrontal regions. These include face and scene-preferential high-level perceptual regions, as well as medial temporal lobe structures, i.e., the perirhinal cortex and hippocampus [96] and the amygdala [97, 98]. We suggest that such effects of vividness and memorability may be directly related to one of the main principles involved in skilled memory: meaningful encoding [40]. However, future functional neuroimaging investigations directly addressing this question are necessary to confirm this hypothesis in our exceptional SMs.

### Associative learning in superior memorizers vs. controls

Two experiments were conducted that involved learning of face-name associations. The first was designed to closely parallel the conditions during international memory competitions, in which arrays of faces with external information and names were presented. Here, as predicted, both SMs excelled, achieving significantly higher scores (but similar learning times) compared to controls. Analyses of fixation patterns generally revealed a large degree of variability, which is attributed to the complexity of the stimuli depicted (arrays of 24 faces), and parallels the variability observed for subjects’ recognition performance. SMs’ fixation patterns did not correlate less with those observed for controls, as compared to when controls’ patterns were correlated. However, both SMs attended to significantly more faces than any of the controls, most probably indicating the use of an optimal non-face specific visual scanning strategy. This is in line with their superior recall performance, and is interpreted as controls fixating fewer faces once their memory capacity was reached.

Based on their own reports and post-experiment debriefing, SMs treated faces as tokens in Experiment 1, using other, non-facial information to elaborate during encoding. Indeed previous research indicates that deeper processing of faces, i.e. when more detailed semantic information is associated with faces during formation of associations, can enhance recognition performance [87, 98–100]. Given the small effect sizes obtained under the use of a confidence measure, some authors have expressed the need for additional studies using recognition tasks to strengthen their ‘preliminary indication of a top-down effect during face learning’ [100]. We believe that SMs’ superior face recall provides strong support for the notion that top-down effects (e.g., via meaningful and elaborate encoding) are beneficial for subsequent recall. However, future studies using a similar experimental design are required to determine whether the underlying mechanism of this advantage is specific to faces (see below).

One could however argue that SMs’ performance in Experiment 1 and the face-name discipline in international championships do not call upon face processing per se, but rather pictorial memory and name recall. However, the observation that both SMs excel in particular in the face-name, but not non-face image learning discipline of the World Memory Championships is not compatible with this idea. Nevertheless, the remaining experiments were designed to expand the initial observations made in Experiment 1, using more controlled stimulus material as used for tasks designed in the domain of face processing (exclusion of non-facial information, equalization of low-level image properties, individual item presentation).

Experiment 2 involved learning of individual face-name associations for same-race (WC) and other-race (EA) faces, all learned with national names. Here, again, in the absence of differences in encoding times, SMs achieved higher scores than control subjects. Importantly, however, all subjects who were not at floor showed comparably superior performance for learning and recalling same-, as compared to other-race face-name associations. These latter results, obtained in the absence of differences in fixation patterns during learning and recall, provide an indication that perceptual face processing does not differ in SMs tested here.

Interestingly, despite SMs outperforming controls in both experiments, controls’ performance was characterized by a high degree of variability. Notably, some controls were able to achieve relatively high scores—especially those who took more time during learning phases. This emphasizes the motivational component underlying superior memory skills as achieved through mnemonic techniques, which can generally improve memory. Motivation, exposure and ability to select and organize information can lead to impressive knowledge displayed by experts across various fields [42]. However, it also calls into question the claim that there are no ‘naturals’ [101]. For instance, Wilding and Valentine [32] reported a case of naturally (i.e., untrained) exceptional memory, whose performance at face-name learning even exceeded those of SMs. Further work is required to determine the nature of untrained superior memory. One possibility is that individuals with natural superior face memory have generally superior face processing abilities, i.e., are Super-recognizers.

### Super face memorizers are not super face recognizers

Perceptual aspects such as distinctiveness of face stimuli or vividness of face imagery have been demonstrated to strongly influence face-name memory [102–105], suggesting that superior memory for face-name associations might rely on exceptional perceptual processing of face stimuli. In experiment 3, both SMs completed three tests, for which data from so-called ‘Super-recognizers’ have been reported [46, 47, 50–52]. These involved two tasks of face recognition (CFMT, BTWF test) and one test of face perception (CFPT). SM1’s scores across all tests were within the range of previously reported normal face processing abilities. SM2 achieved normal scores on the CFMT, the test most commonly used for identification of SRs and developmental prosopagnosics [46, 47, 50–52]. For the CFPT and BTWF test, SM2 achieved somewhat higher scores, which were, however, in the high end of the data previously reported for normal controls [46, 47]. Thus, based on these previously utilized tests of superior face recognition, the SMs tested here cannot be considered as Super-recognizers.

### Normal perceptual face processing in superior memorizers

Experiments 4 and 5 were conducted to further verify the absence of perceptual processing differences in SMs. Importantly, these experiments did not require learning of face-name associations, but instead involved old-new recognition of same and other-race faces, and delayed matching of upright and inverted faces. In Experiment 4 both SMs showed an advantage for discriminating between previously seen and unseen same- over other-race faces. Importantly, as in Experiment 2, the magnitude of this so-called other-race effect did not differ from that observed in controls, and all observers showed comparable patterns of fixations. Finally, the results of Experiment 5, which revealed inversion–related performance detriments of comparable magnitude for normal observers and SMs, provided further support of the idea that perceptual face processing is uninfluenced by memory techniques. An open question, however, is whether perceptual face processing as tested here is robust to feedback training reported to impact face matching performance [106, 107].

### Conclusion

Our data shows that SMs’ superior performance in face-name association is not accompanied by superior/expert visual skills. It also indicates that SMs excel at face (and other visual) memory tasks because of deliberate training in mnemonic strategies, rather than a pre-existing or acquired propensity in processing facial information or a reorganization of the computational processes of the face system. In other words, exceptional memorizers are made, not born [96] and do not possess exceptional visual abilities. In fact, despite extensive training lending to their ranking as leading competitors in the face memory discipline of the World Memory Championships, the SMs tested here did not differ from control subjects in tests of perceptual face processing or face recognition and cannot be considered ‘Super-recognizers’ [46]. However, they did show superior performance when tested with the experimental designs used during international competitions, which involved the memorization of the simultaneous presentation of many (uncontrolled) faces and names. Future studies are required to clarify the nature of this advantage, including its neuro-functional basis. Furthermore, it would be interesting to investigate in detail SMs who, despite extensive training, are particularly challenged by face memory disciplines, as well as testing Super-recognizers with the experiments reported here. Lastly, it would also be of interest to determine whether, during acquisition of mnemonic techniques, their learning curves for faces (as compared to other visual stimuli) would differ qualitatively from those of normal controls given their presumed higher efficiency at perceptual face processing and face memory. This would indicate whether stimulus-specific training effects can be found provided superior pre-training proficiency at handling certain types of visual information.

Taken together, our results support the idea that certain aspects of face processing are unaffected by explicit training with mnemonic techniques as employed by SMs. These rely mostly on perceptual mechanisms which are possibly solely modulated through repeated, real-life experience [108–111]. Although our findings are confined within the domain of face processing, they are relevant for theories of memory and perception as they indicate that exceptional memory can be observed in the absence of exceptional visual processing abilities. The neural computations performed by the visual system might be robust and impermeable to the computations performed by the mnemonic system.

## Supporting Information

S1 Fig Individual fixation maps for Experiment 2—Western Caucasian and Eastern Asian face-name learning. Note that SMs’ fixation patterns are in the range of those observed for controls. For example SM1’s pattern with WC faces is very similar to that of Control 1 (top-left).
(PDF)

S2 Fig Individual fixation maps for Experiment 4—Other-race effect (ORE). Note that SMs’ fixation patterns are in the range of those of the controls. For example SM2’s pattern with WC faces is very similar to that of Control 8 (second row, forth column).
(PDF)

S1 Table Results of the cluster analyses for Experiments 2 and 3. The ZR scores correspond to the Z Fisher’s tranformed correlations between the individual fixation maps and the prototypical local and global models (see Methods).
(DOCX)
