# Supplementary figures and images for: Super-Memorizers Are Not Super-Recognizers
Source: PLoS One. 2016 Mar 23;11(3):e0150972. doi: 10.1371/journal.pone.0150972 (PMC4805230; doi:10.1371/journal.pone.0150972)

# Western Caucasian face-name learning

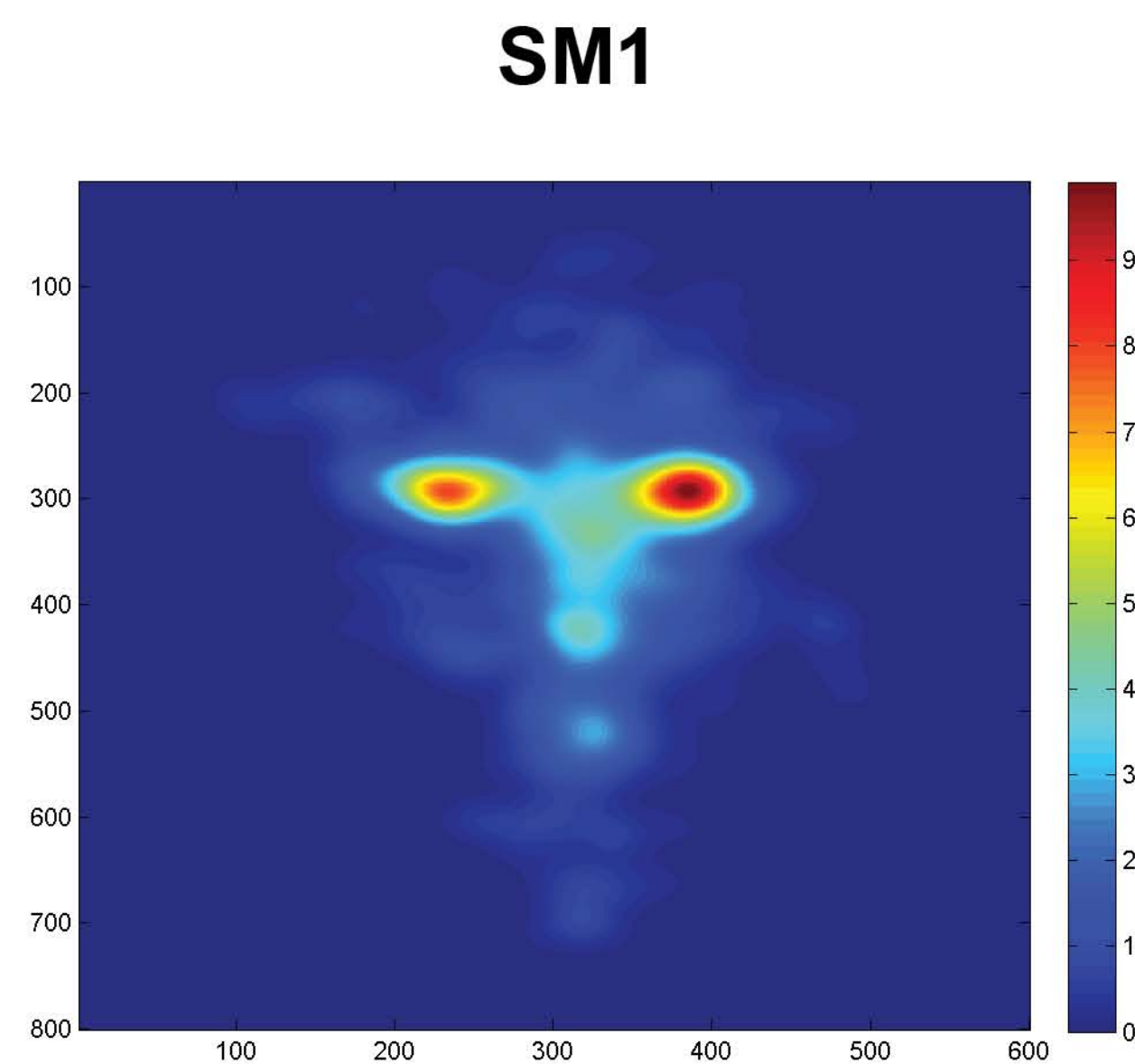

# Eastern Asian face-name learning

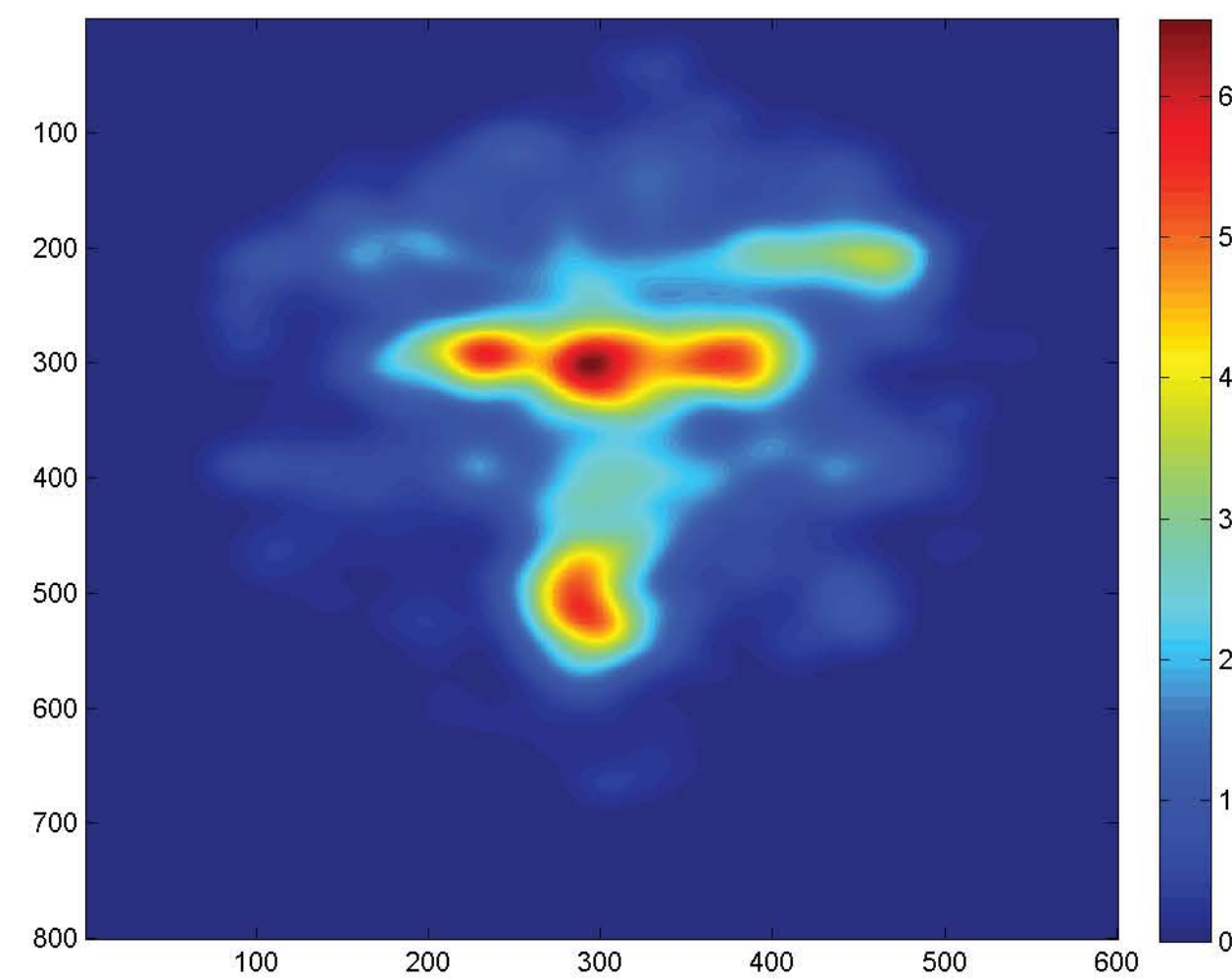

SM1

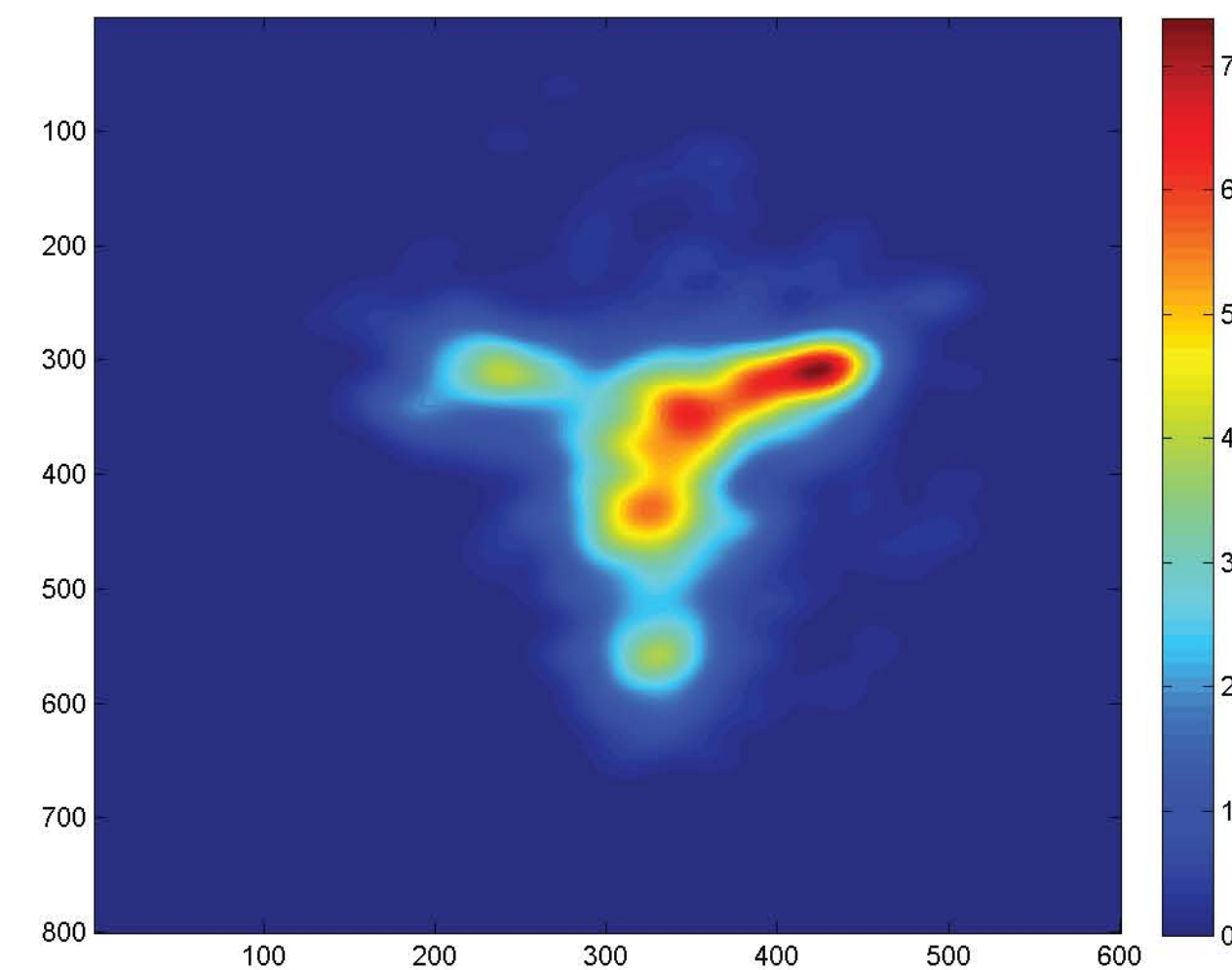

## SM2

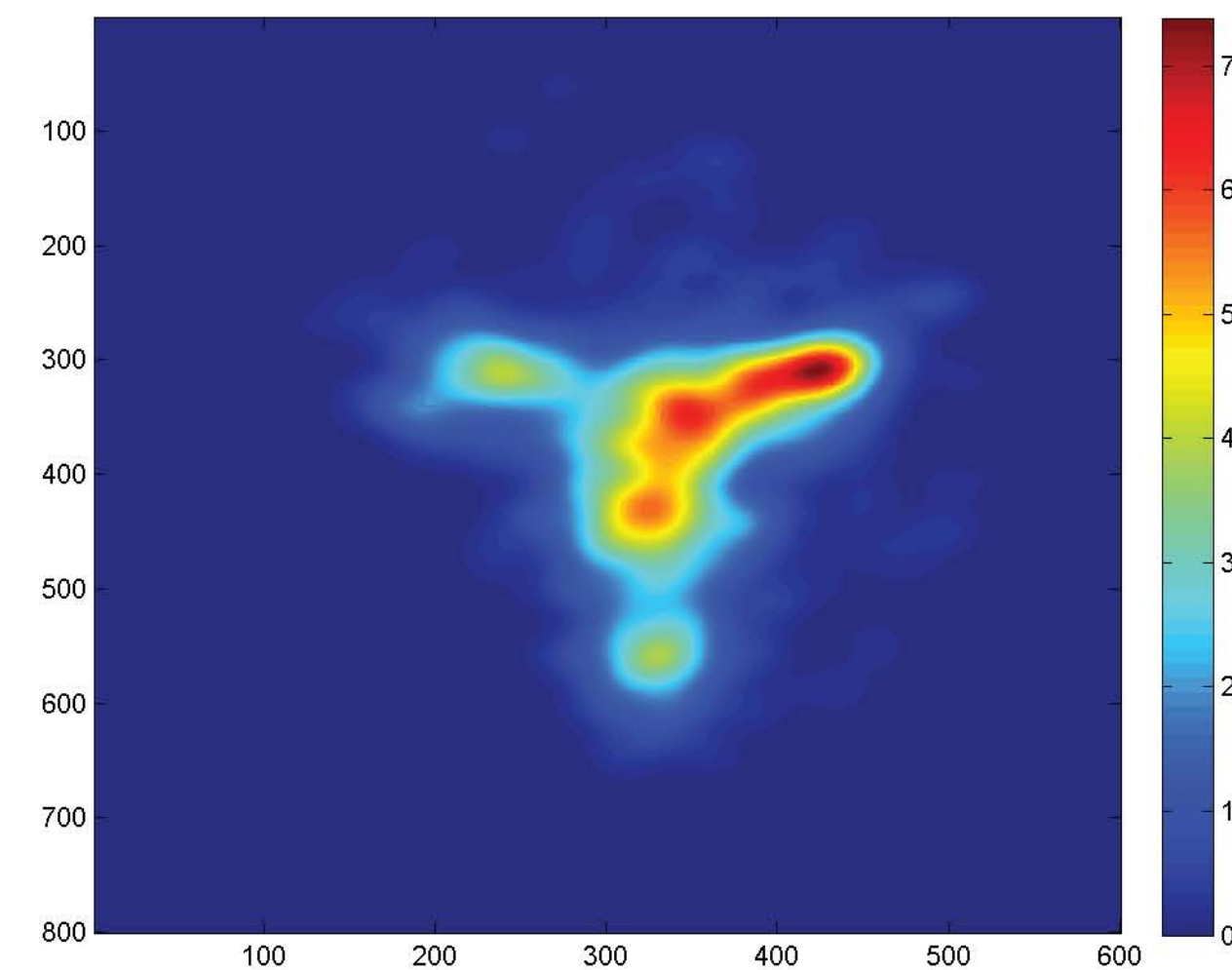

## Controls

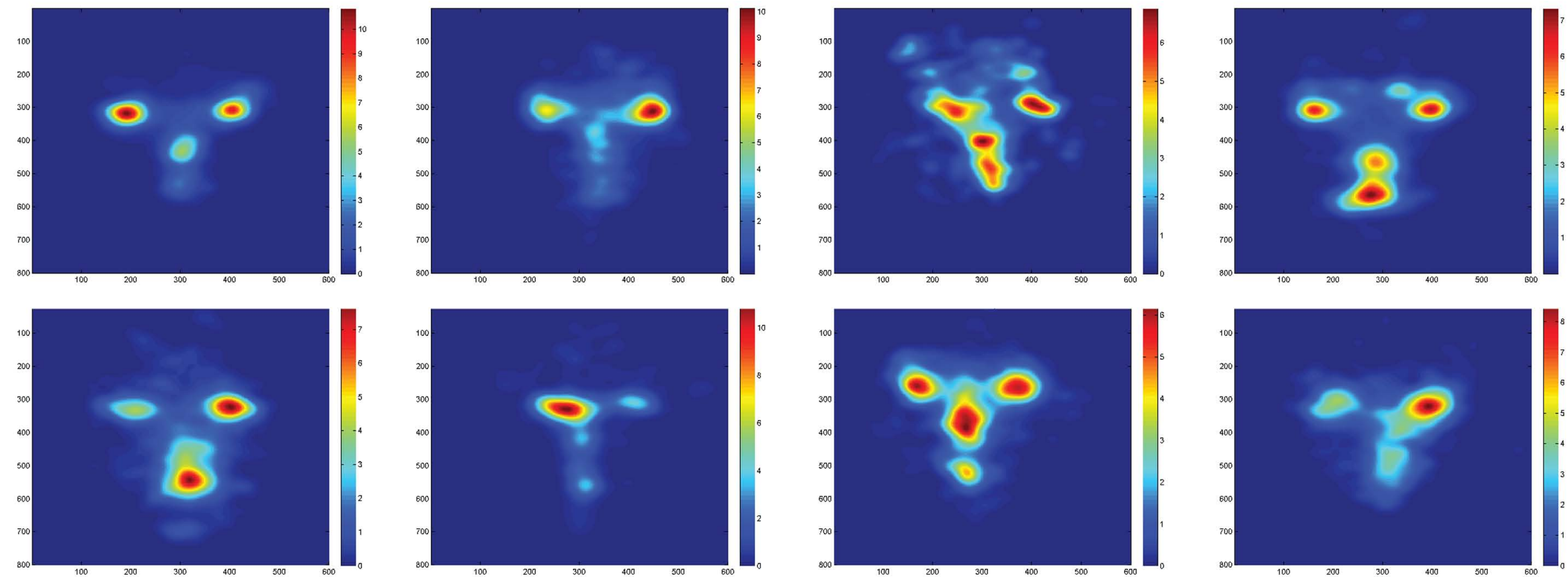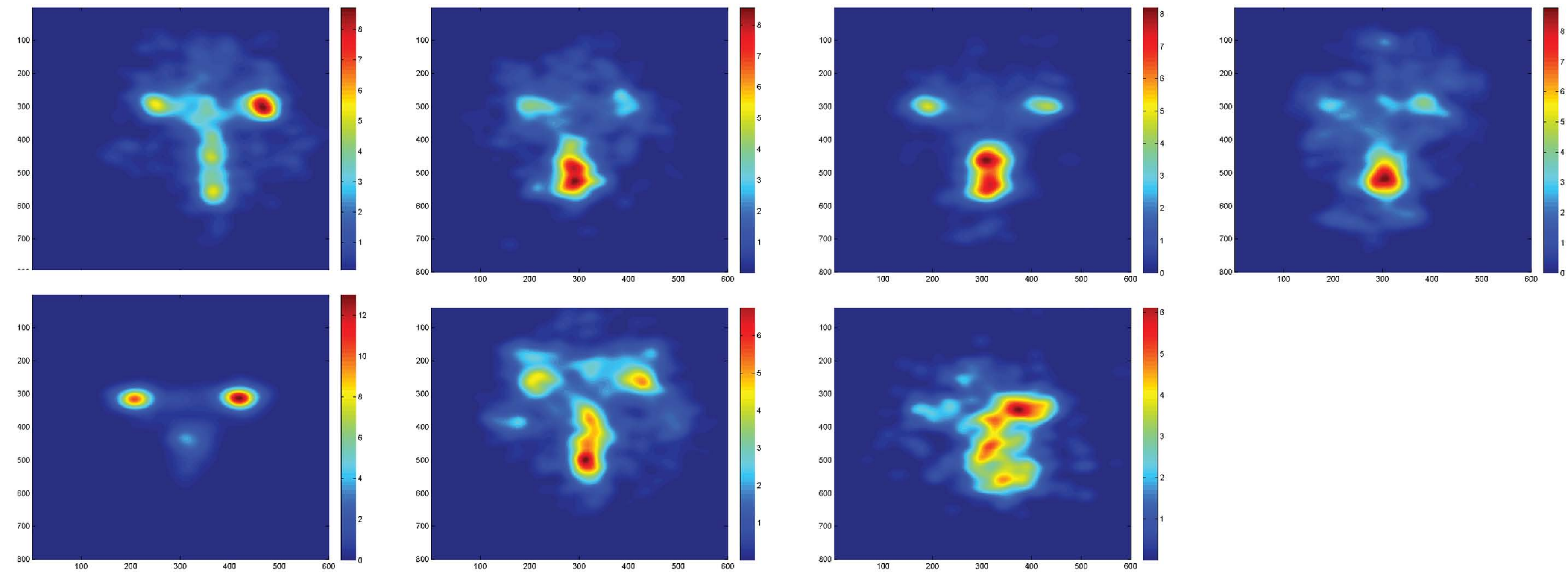

Supplement: S1 Fig — Note that SMs’ fixation patterns are in the range of those observed for controls. For example SM1’s pattern with WC faces is very similar to that of Control 1 (top-left). (PDF) [file pone.0150972.s001.pdf]

SM1

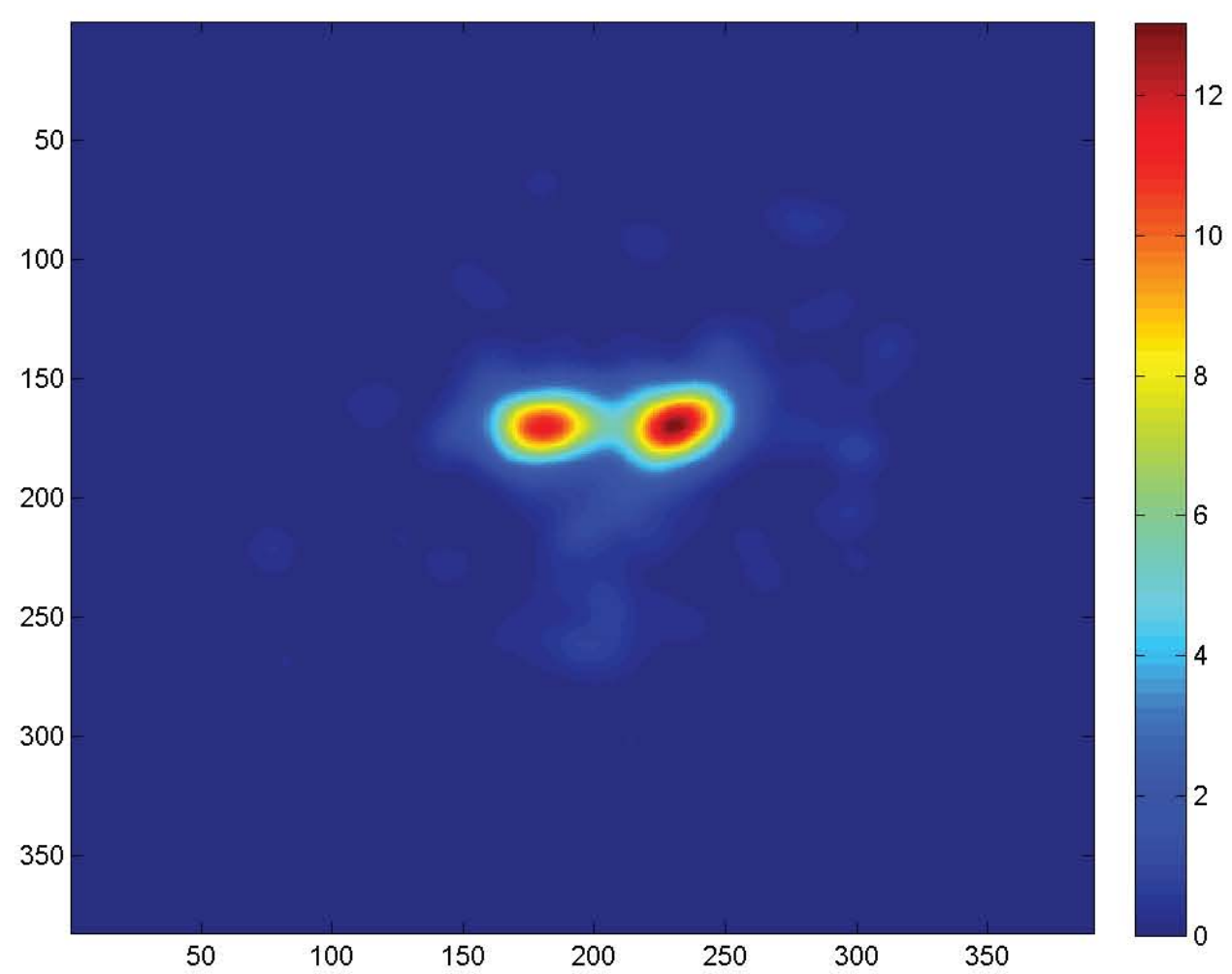

## SM2

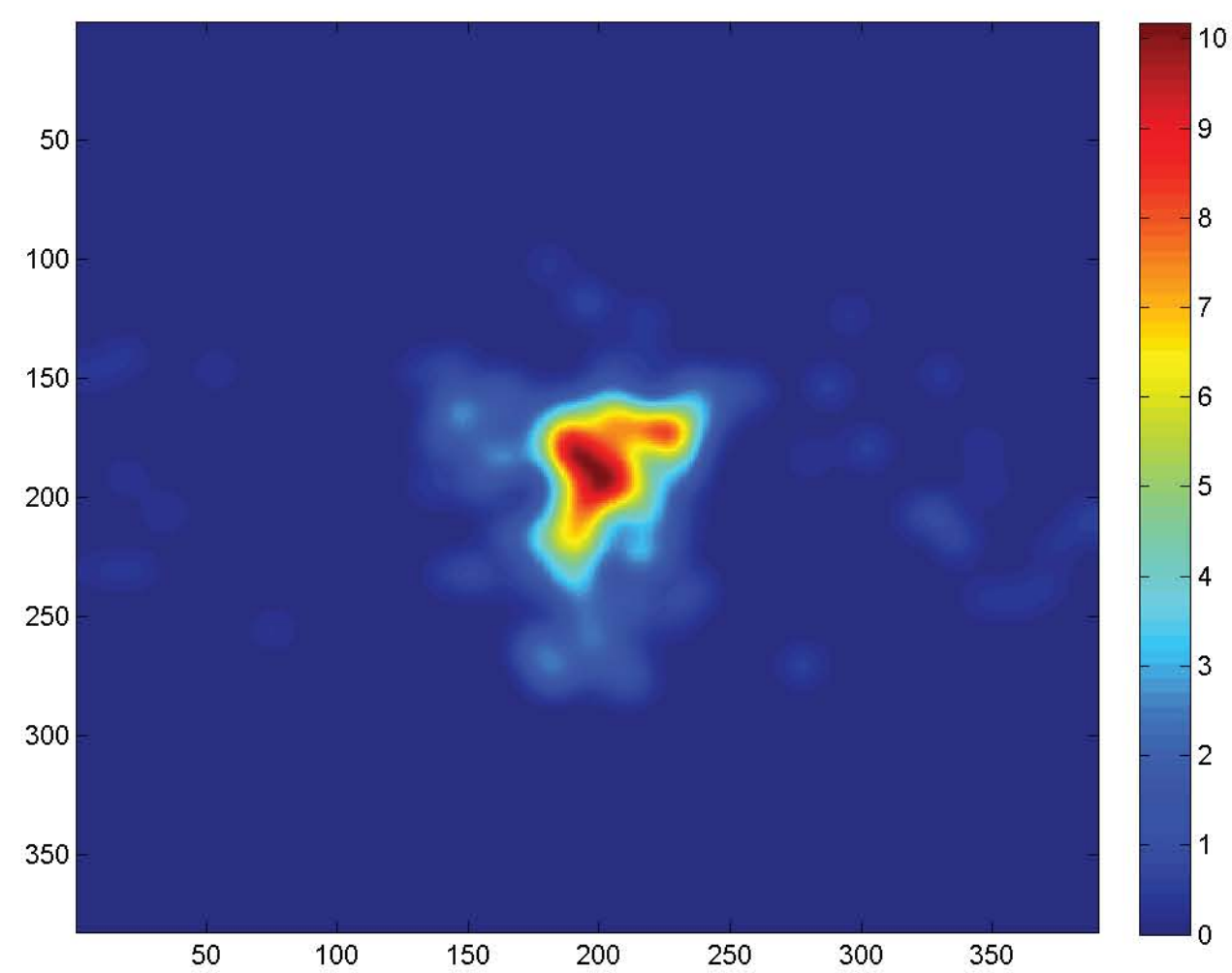

## Controls

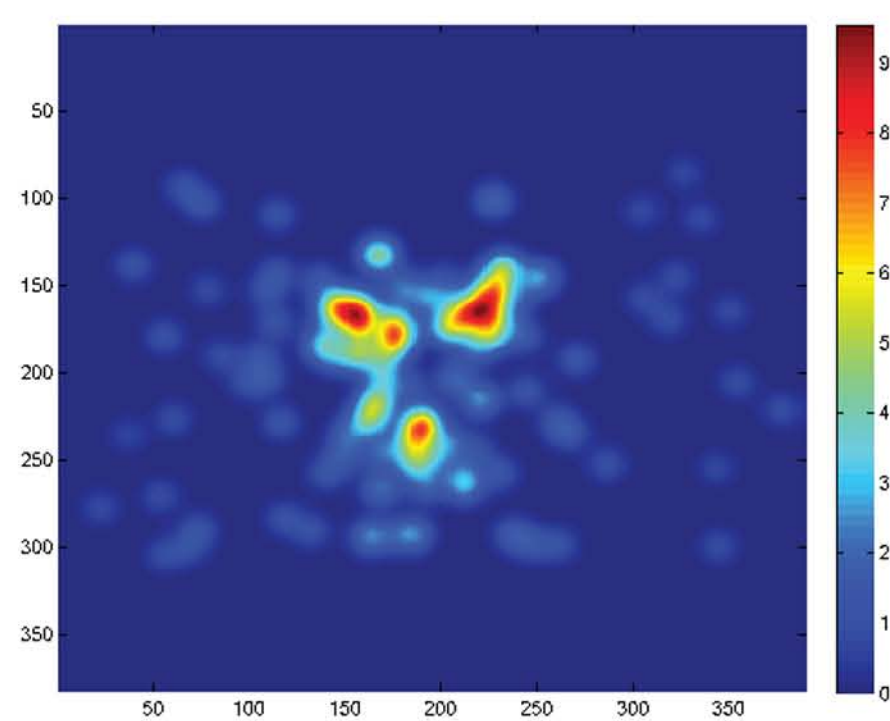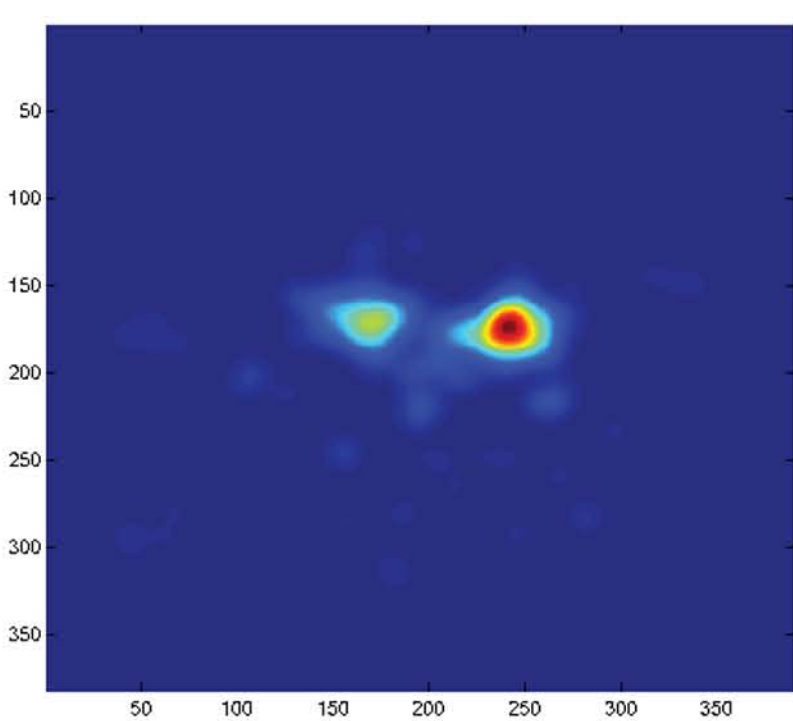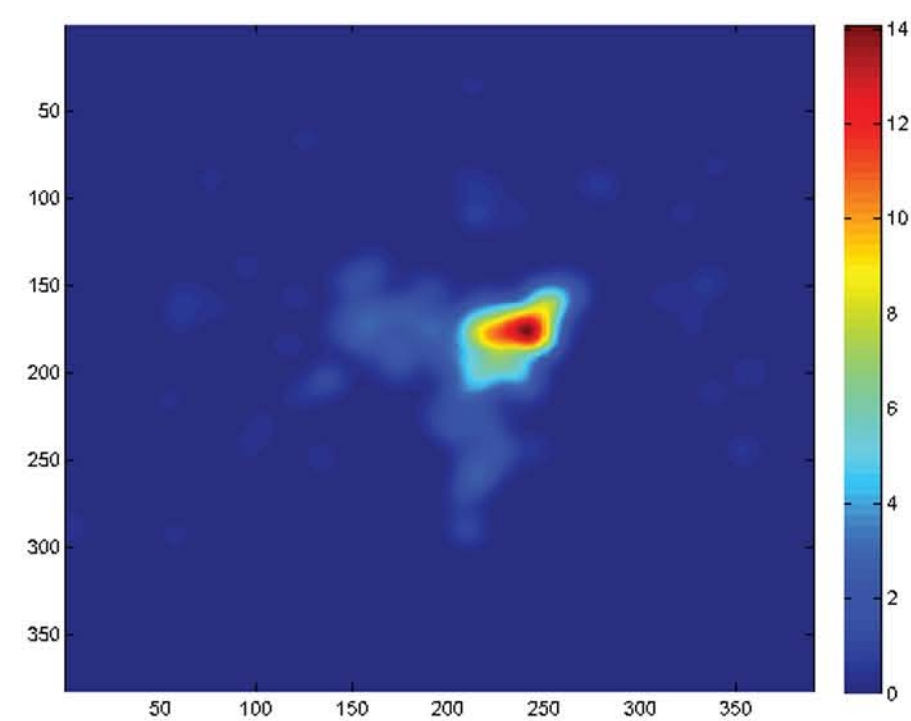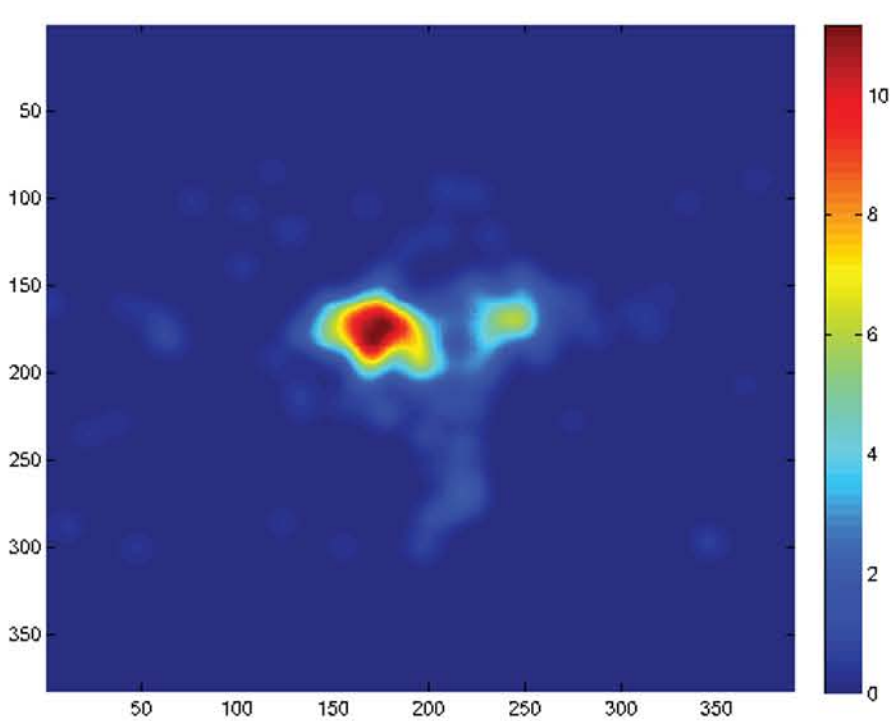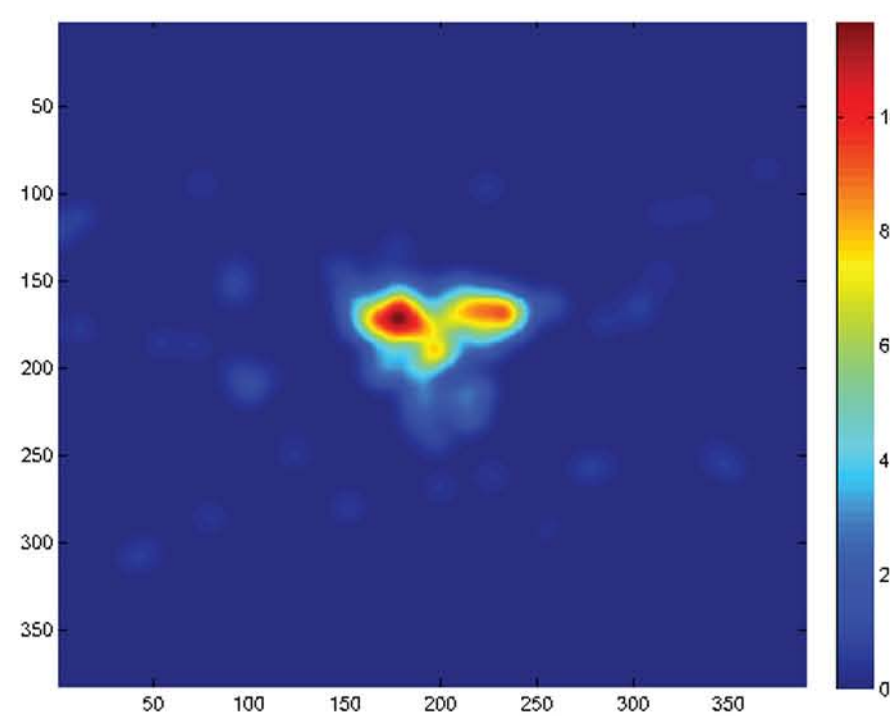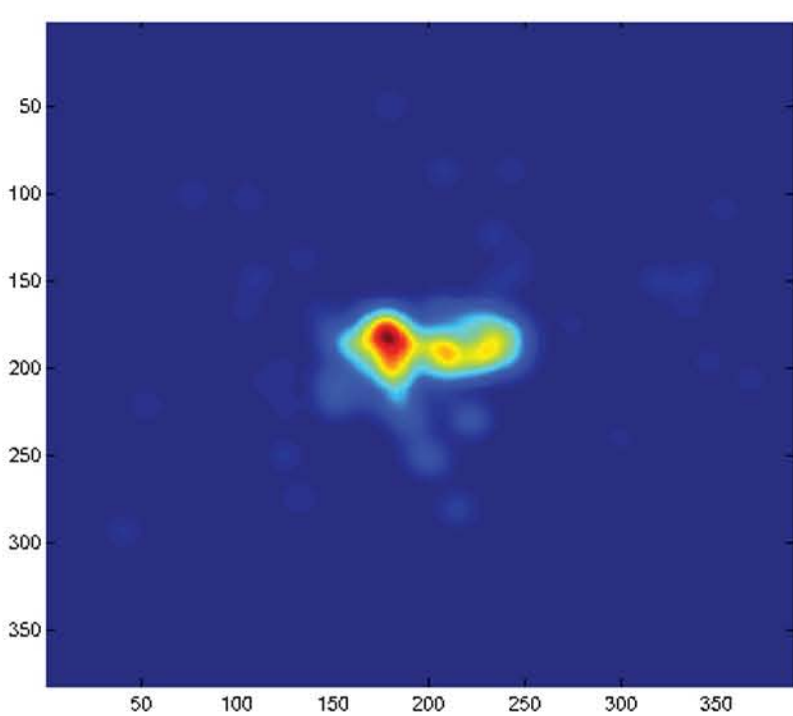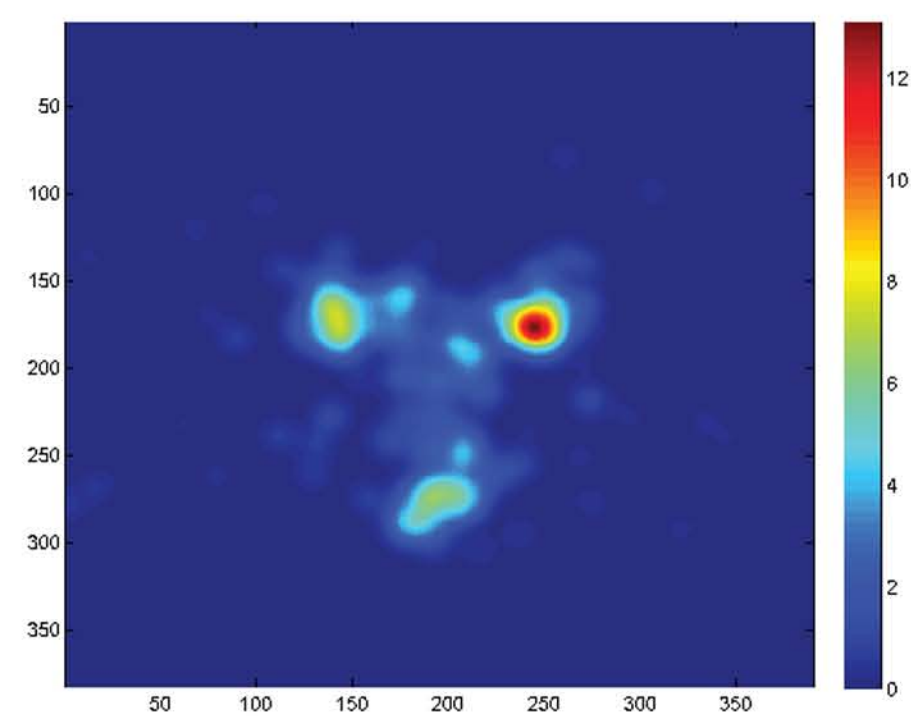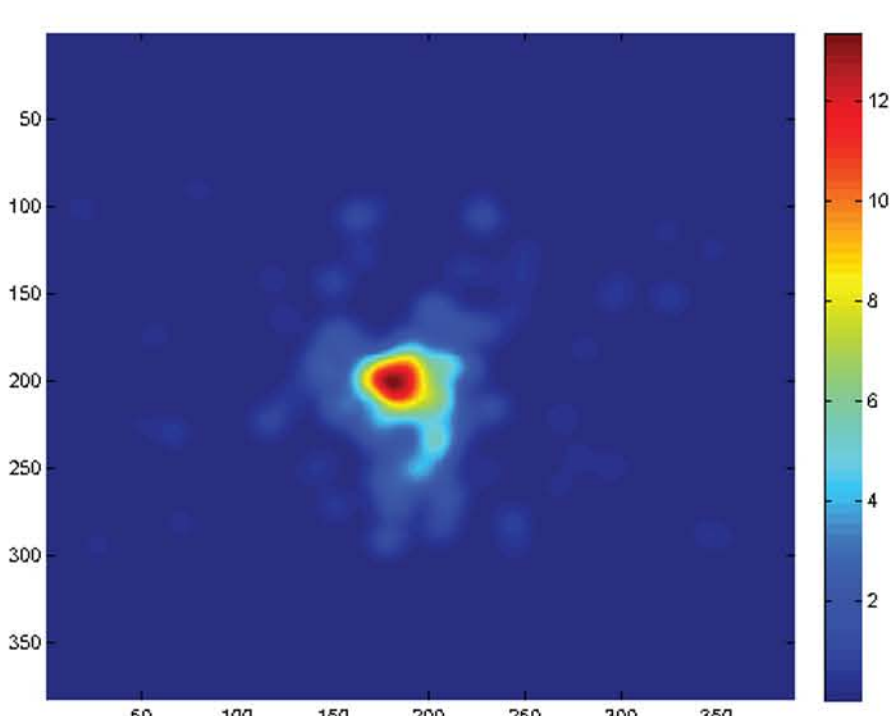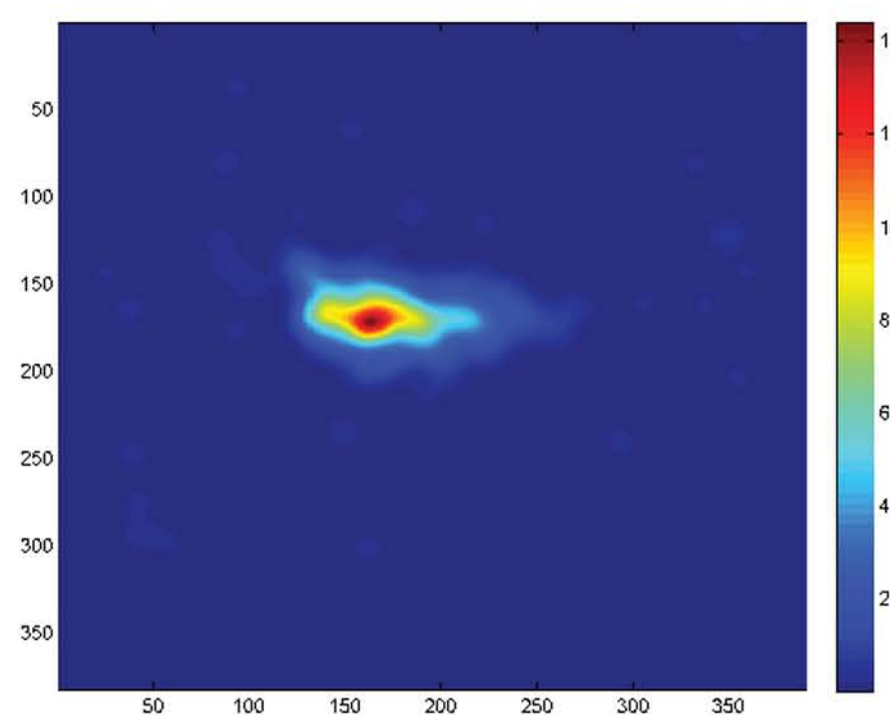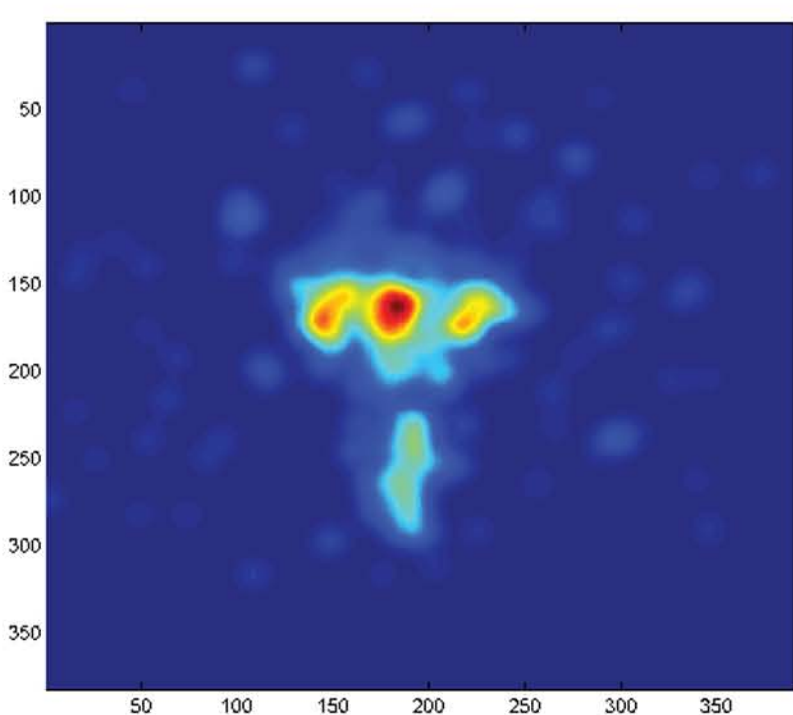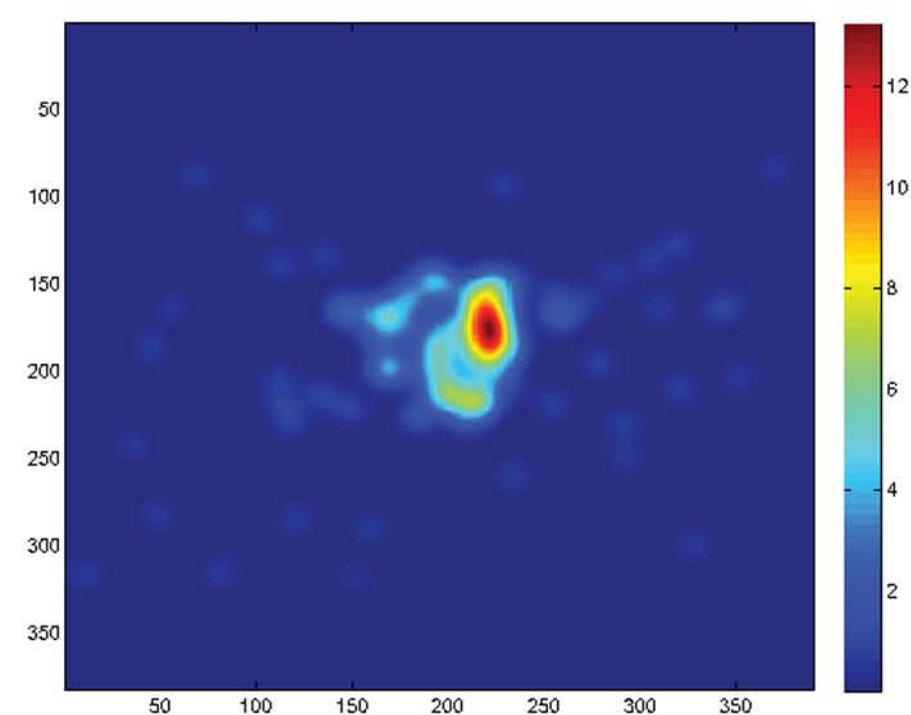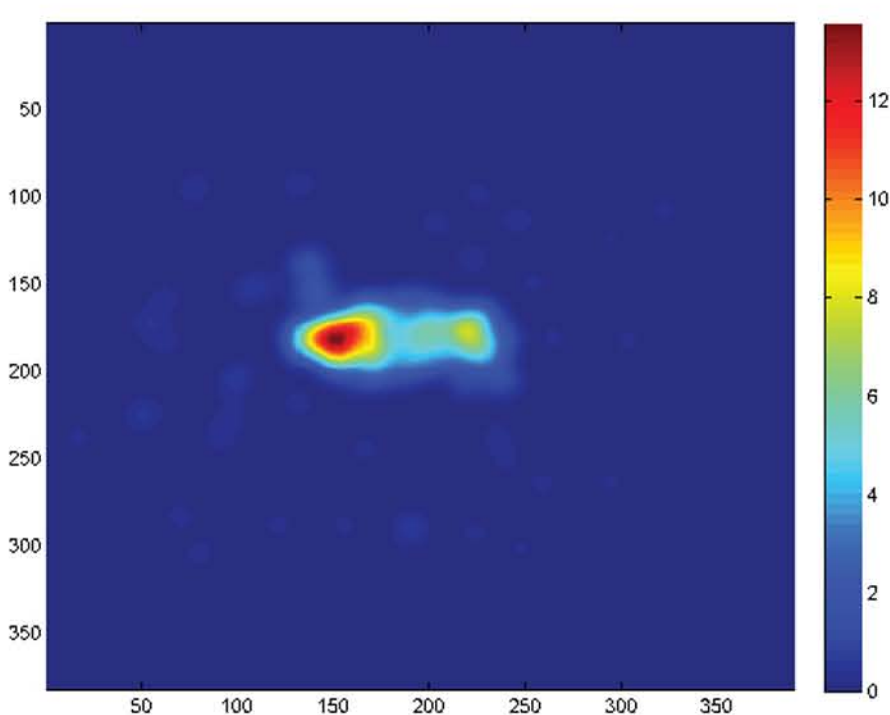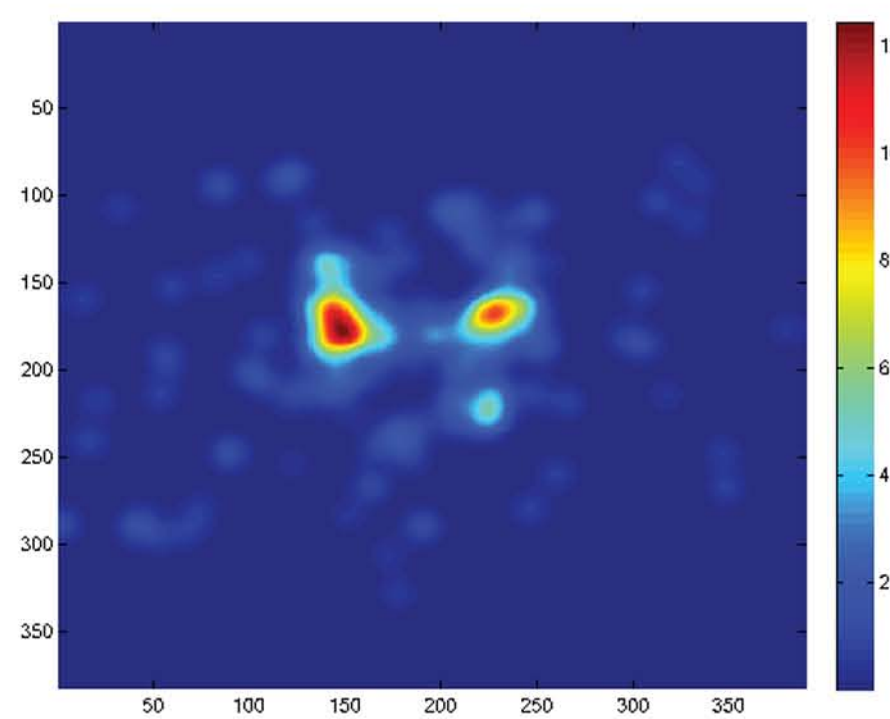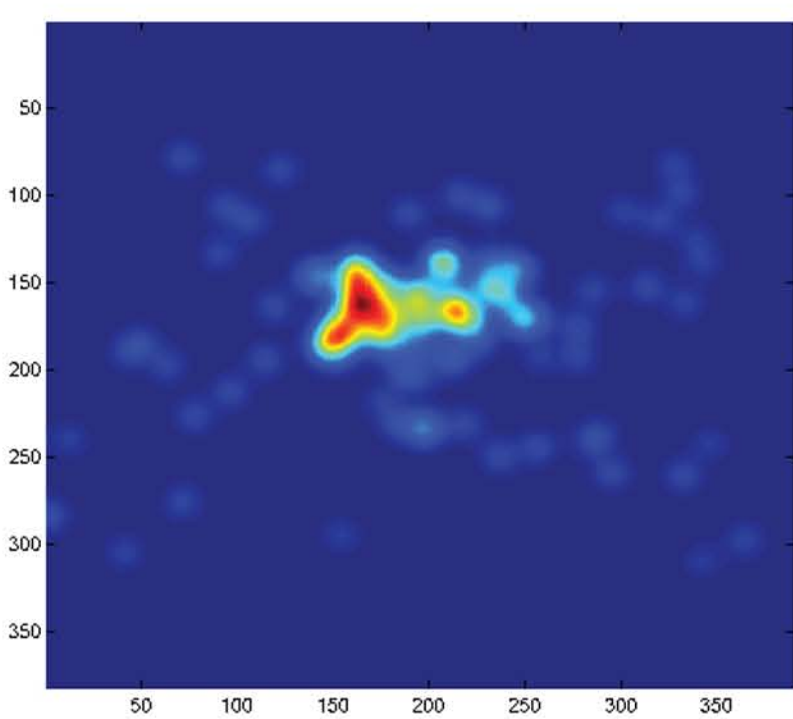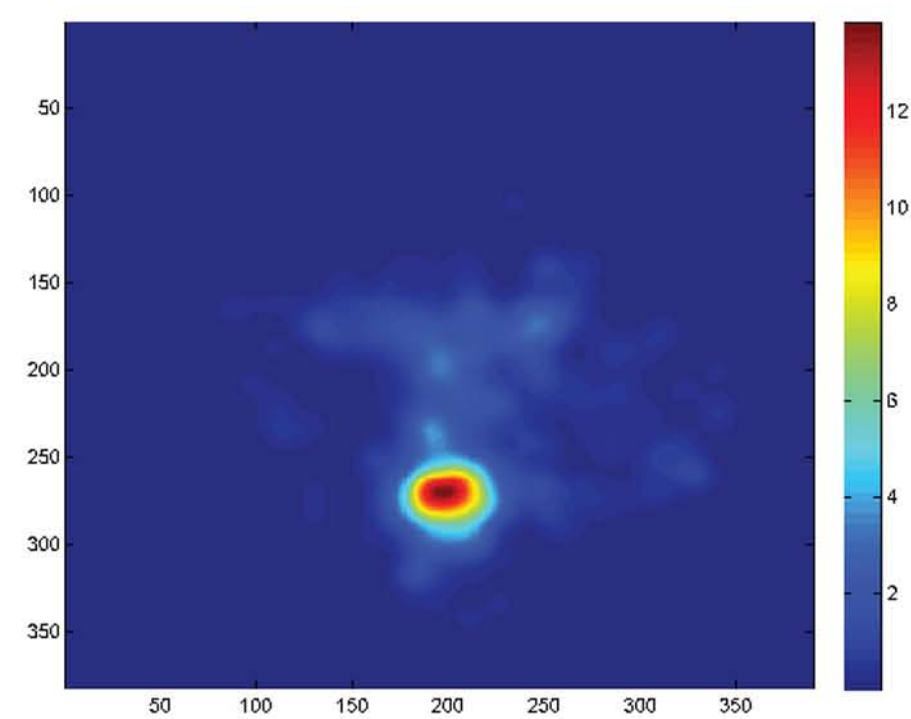

Supplement: S2 Fig — Note that SMs’ fixation patterns are in the range of those of the controls. For example SM2’s pattern with WC faces is very similar to that of Control 8 (second row, forth column). (PDF) [file pone.0150972.s002.pdf]
