# Supplementary material for: Super-Memorizers Are Not Super-Recognizers
Source: PLoS One. 2016 Mar 23;11(3):e0150972. doi: 10.1371/journal.pone.0150972 (PMC4805230; doi:10.1371/journal.pone.0150972)
Supplement: S1 Table — The ZR scores correspond to the Z Fisher’s tranformed correlations between the individual fixation maps and the prototypical local and global models (see Methods). (DOCX) [file pone.0150972.s003.docx]

|  |  | ZR to Local model | ZR to Global model |
| --- | --- | --- | --- |
| Experiment 2 | | | |
| WC | **SM1** | .58 | .37 |
|  | **SM2** | .58 | .62 |
|  | C1 | .82 | .48 |
|  | C2 | 1.04 | .40 |
|  | C3 | .55 | .39 |
|  | C4 | .51 | .59 |
|  | C5 | .39 | .35 |
|  | C6 | .66 | .25 |
|  | C7 | .87 | .46 |
|  | C8 | .74 | .38 |
| EA | **SM1** | .38 | .29 |
|  | **SM2** | .59 | .41 |
|  | C1 | .36 | .42 |
|  | C2 | .68 | .52 |
|  | C3 | .35 | .42 |
|  | C4 | .56 | .43 |
|  | C5 | .23 | .08 |
|  | C6 | .59 | .37 |
|  | C7 | .74 | .21 |
| Experiment 3 | | | |
|  | **SM1** | .47 | .27 |
|  | **SM2** | .42 | .86 |
|  | C1 | .47 | .32 |
|  | C2 | .38 | .58 |
|  | C3 | .70 | .47 |
|  | C4 | .51 | .23 |
|  | C5 | .66 | .30 |
|  | C6 | .69 | .47 |
|  | C7 | .46 | .18 |
|  | C8 | .33 | 1.13 |
|  | C9 | .54 | .41 |
|  | C10 | .99 | .39 |
|  | C11 | .55 | .39 |
|  | C12 | .36 | .66 |
|  | C13 | .56 | .22 |
|  | C14 | .38 | .45 |
|  | C15 | .69 | .55 |
